# Supplementary material for: Ubiquitous selection for mecA in community-associated MRSA across diverse chemical environments
Source: Nat Commun. 2020 Nov 27;11:6038. doi: 10.1038/s41467-020-19825-3 (PMC7695840; doi:10.1038/s41467-020-19825-3)
Supplement: Supplementary file 1 — Supplementary Information [file 41467_2020_19825_MOESM1_ESM.pdf]

Supplementary Information

Ubiquitous selection for *mecA* in community-associated MRSA across diverse chemical environments

Snitser et al.

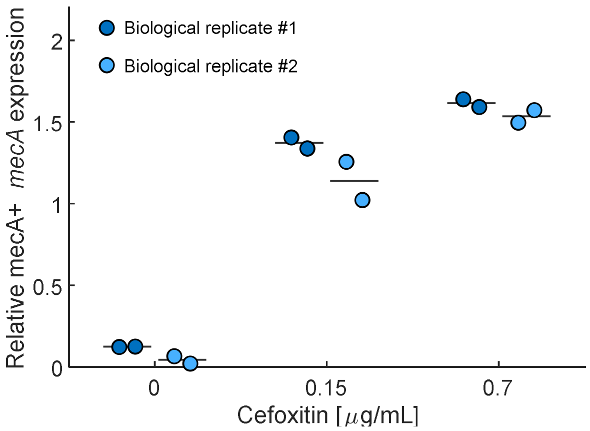

Supplementary Figure 1: Cefoxitin induces *mecA* expression. Quantitative reverse transcription real-time PCR (qRT-PCR) analysis of *mecA* gene with and without supplementing the medium with cefoxitin. *gmk* was used as a reference gene for normalization of qRT-PCR data. Data points represent two technical replicates (same-colored dots) of each of the two biological replicates (dark and light blue colored dots), for each cefoxitin concentration; black horizontal lines represent the mean relative *mecA* expression of every two technical replicates. Combining the biological and technical replicates, 0.15 µg/mL cefoxitin elevated *mecA* mRNA expression by 15.0, and 0.7 µg/mL cefoxitin elevated *mecA* mRNA expression by 18.9. Source data are provided as a Source Data file.

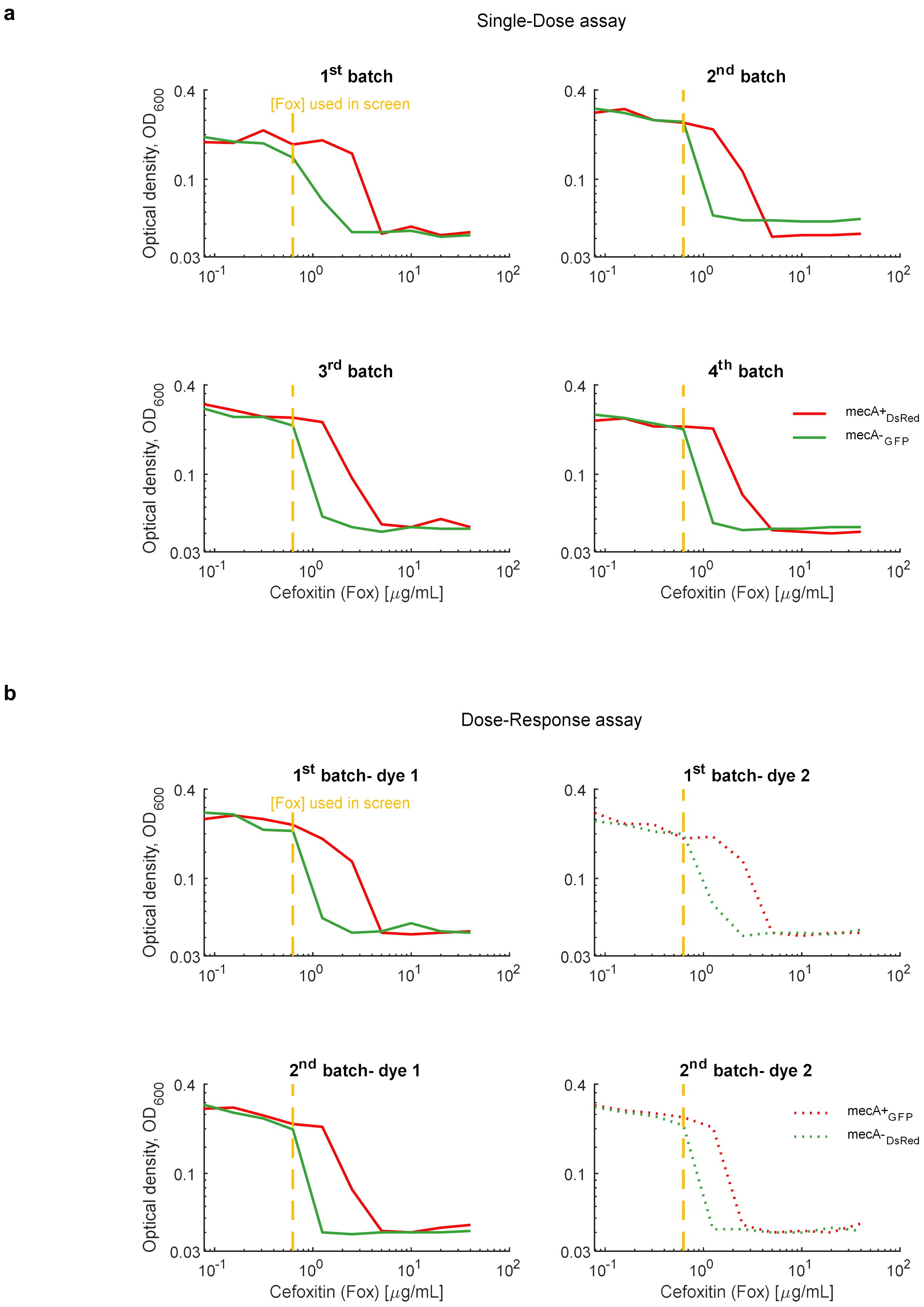

Supplementary Figure 2: Pre-induction assays (done before each screen batch). Growth of diluted 1:100 aliquoted bacterial cultures, on a cefoxitin gradient for ~4h, for pre- induction before each **a,** Single-Dose assay, and **b,** Dose-Response assay. Solid lines represent mecA+ (DsRed) and mecA- (GFP) strains. Dotted lines represent the dye-swap strains, mecA+ (GFP) and mecA- (DsRed). Yellow dashed line represents the cefoxitin concentration chosen for the screen.

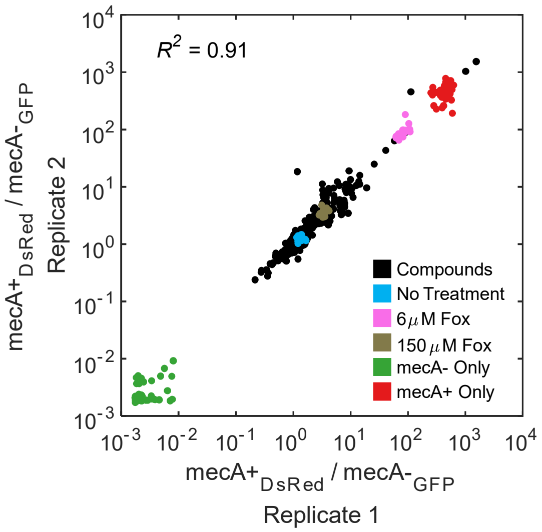

Supplementary Figure 3: Assay is highly reproducible. Log10-ratios of mecA+ (DsRed) to mecA- (GFP) cells fluorescence of two same-day replicate experiments (Pilot Screen, Methods) are plotted against one another (linear regression R^2=0.91).

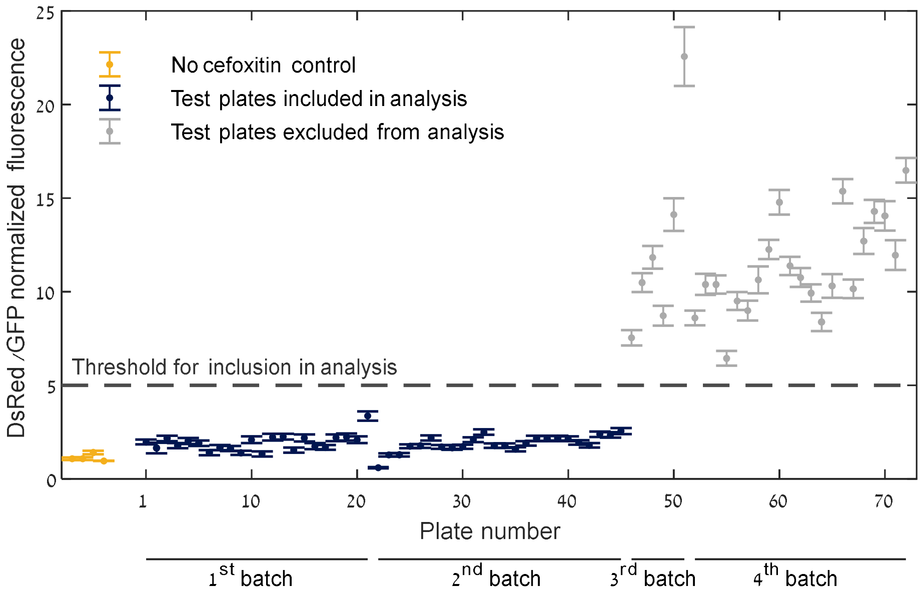

Supplementary Figure 4: Single-Dose test plates excluded from analysis. Median of DsRed to GFP fluorescent signal in 'No Treatment' control (not treated wells, control for no selection for *mecA*, Methods) across all plates from all individual sub-screen batches of the Single-Dose assay compared to no cefoxitin control plates. Error bars are standard errors of the mean. Ratio of DsRed/GFP > 5 was set as a threshold for inclusion of a plate in the analysis (Number of 'No Treatment' control wells for test plates 1,3-22, n = 16; for test plates 23-73 and for 'no cefoxitin' control plates 1-3, n = 22; for test plate 2, n = 8; for 'no cefoxitin' control plate 4, n = 150). Source data are provided as a Source Data file.

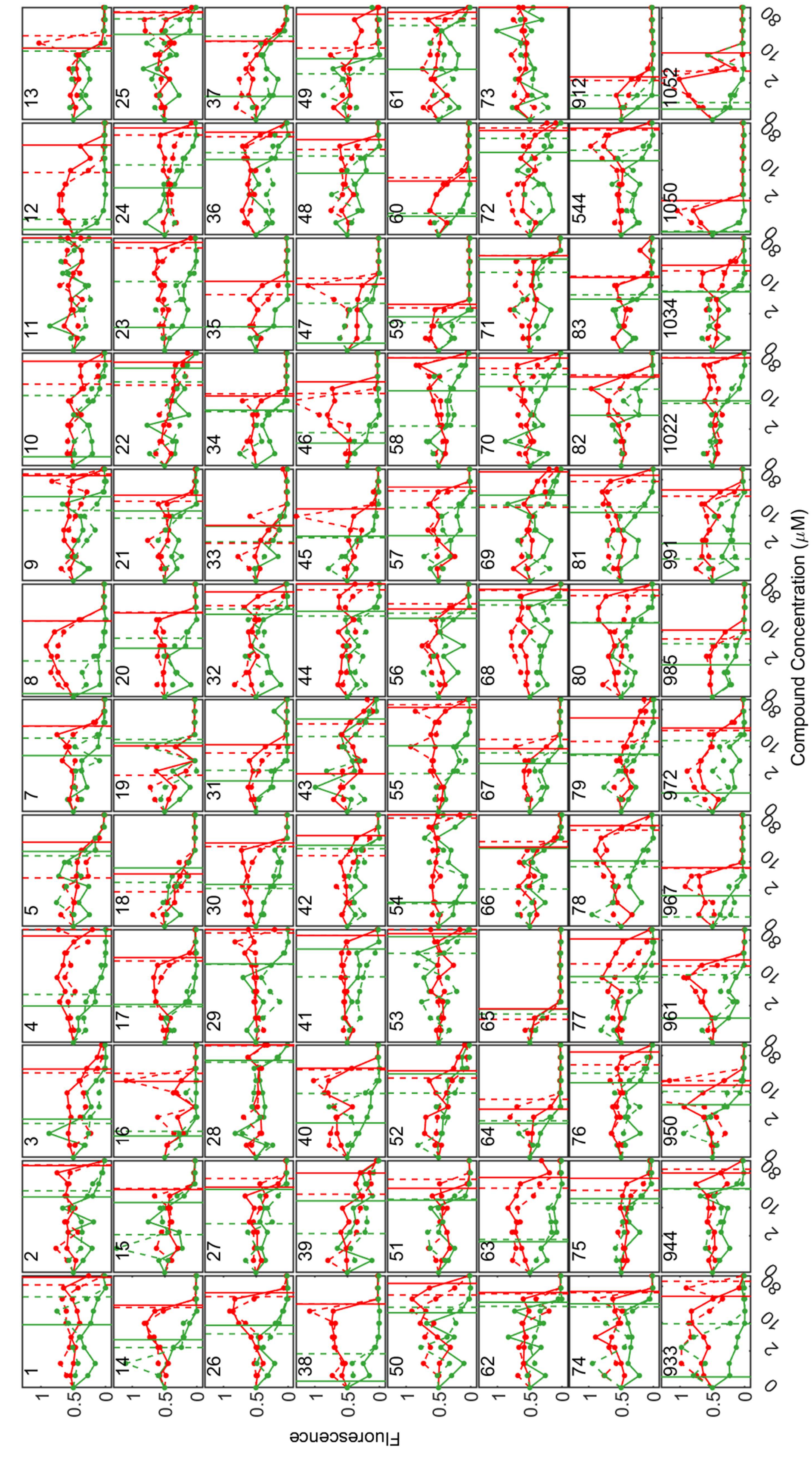

Supplementary Figure 5: Dose response curves and IC_{50}'s of all 1990 compounds screened in Dose-Response assay (1/22).

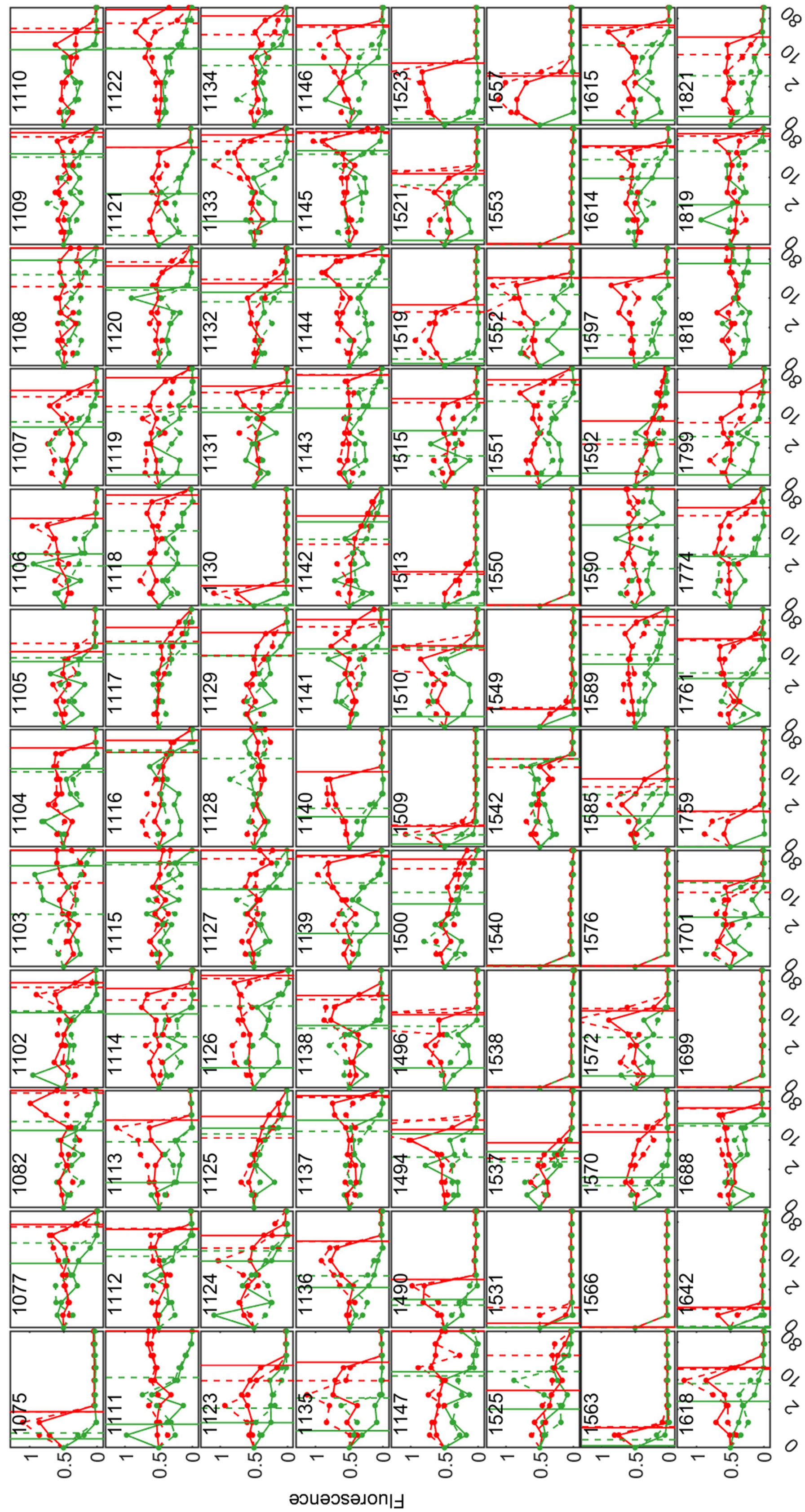

Supplementary Figure 5: Dose response curves and IC_{50}'s of all 1990 compounds screened in Dose-Response assay (2/22).

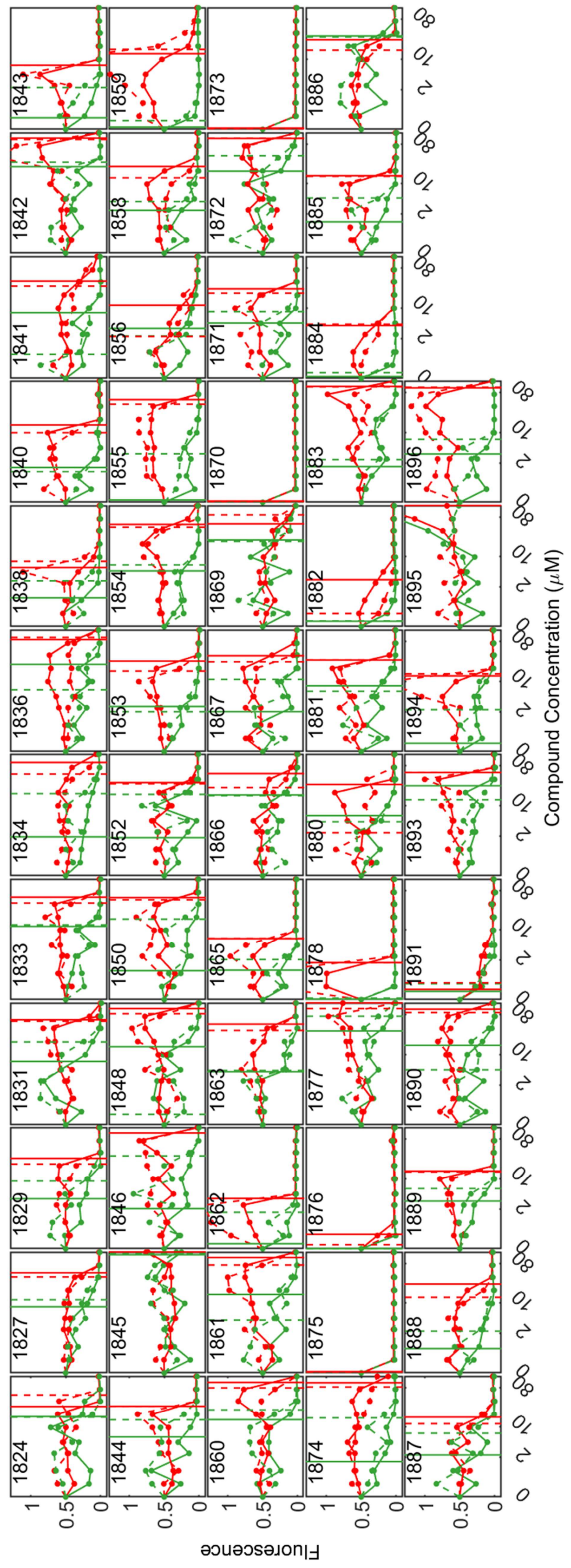

Supplementary Figure 5: Dose response curves and IC_{50}'s of all 1990 compounds screened in Dose-Response assay (3/22).

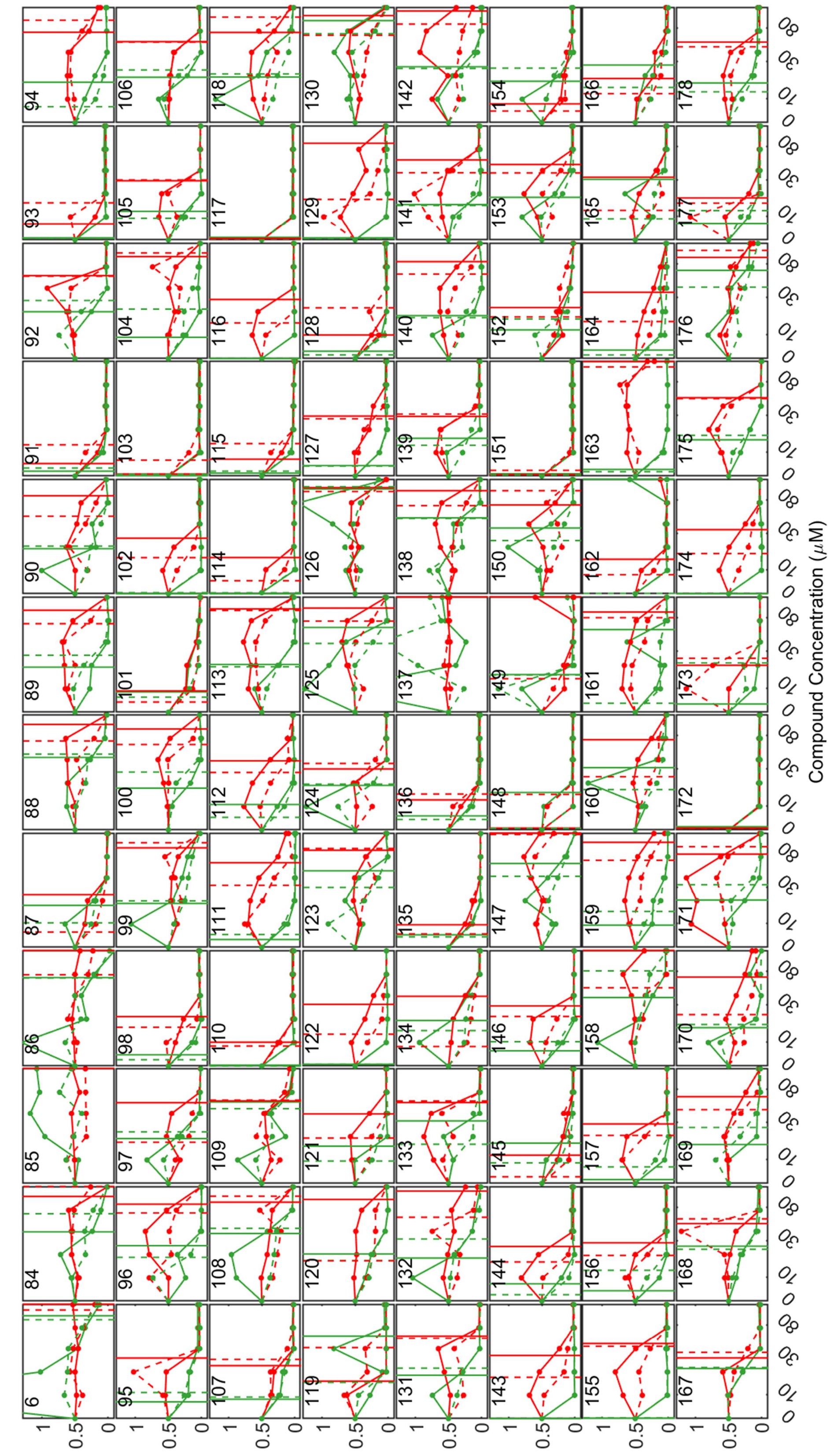

Supplementary Figure 5: Dose response curves and IC_{50}'s of all 1990 compounds screened in Dose-Response assay (4/22).

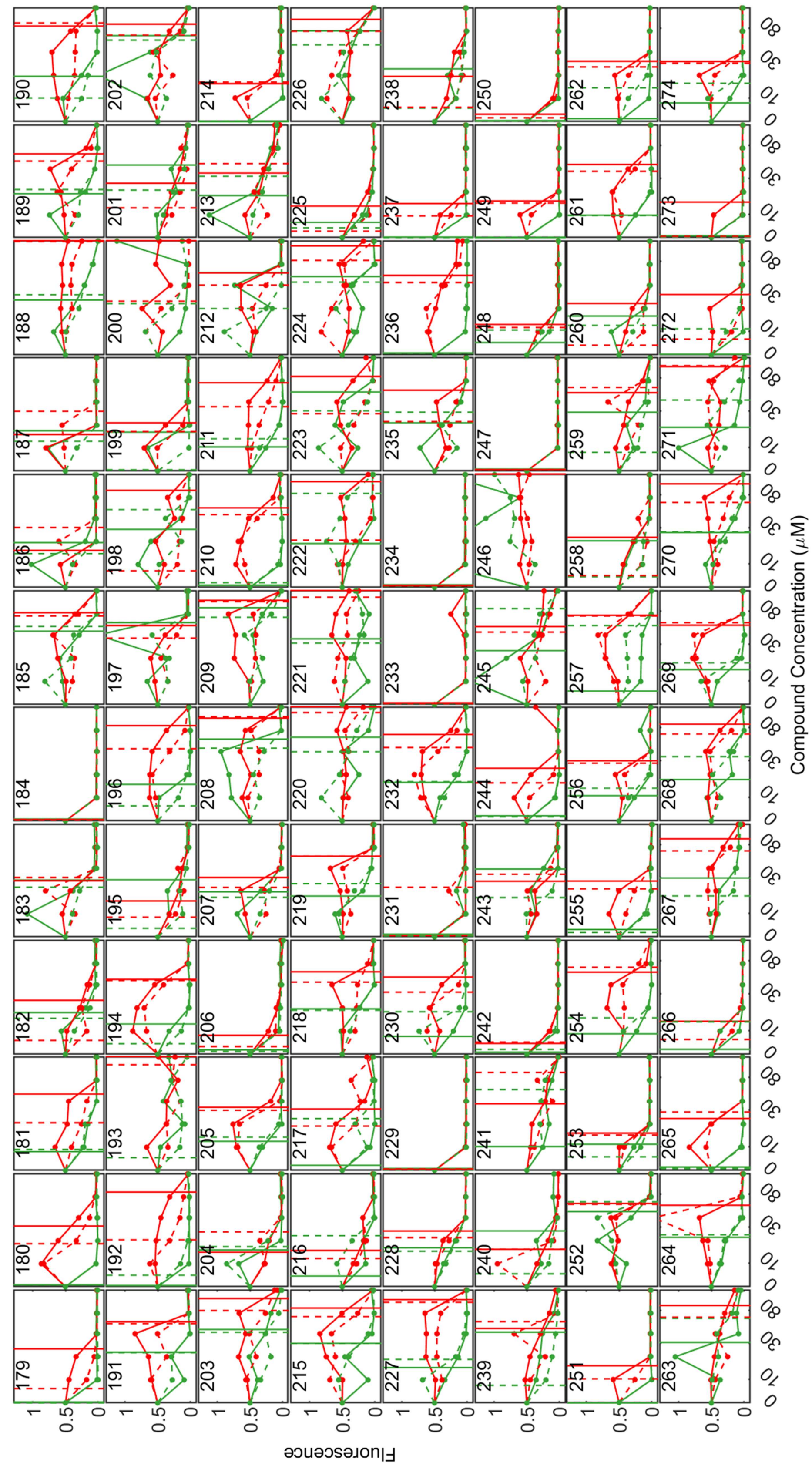

Supplementary Figure 5: Dose response curves and IC_{50}'s of all 1990 compounds screened in Dose-Response assay (5/22).

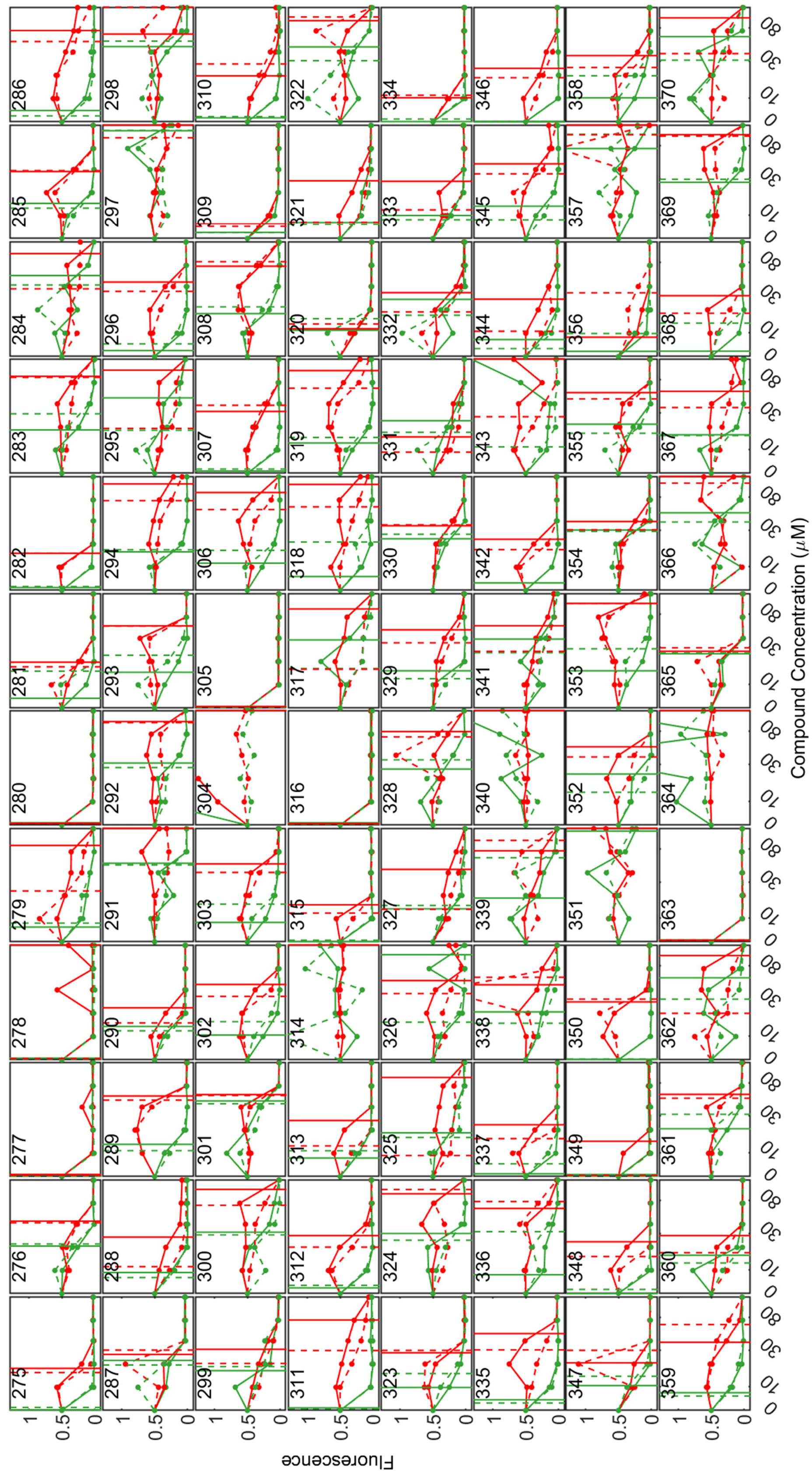

Supplementary Figure 5: Dose response curves and IC_{50}'s of all 1990 compounds screened in Dose-Response assay (6/22).

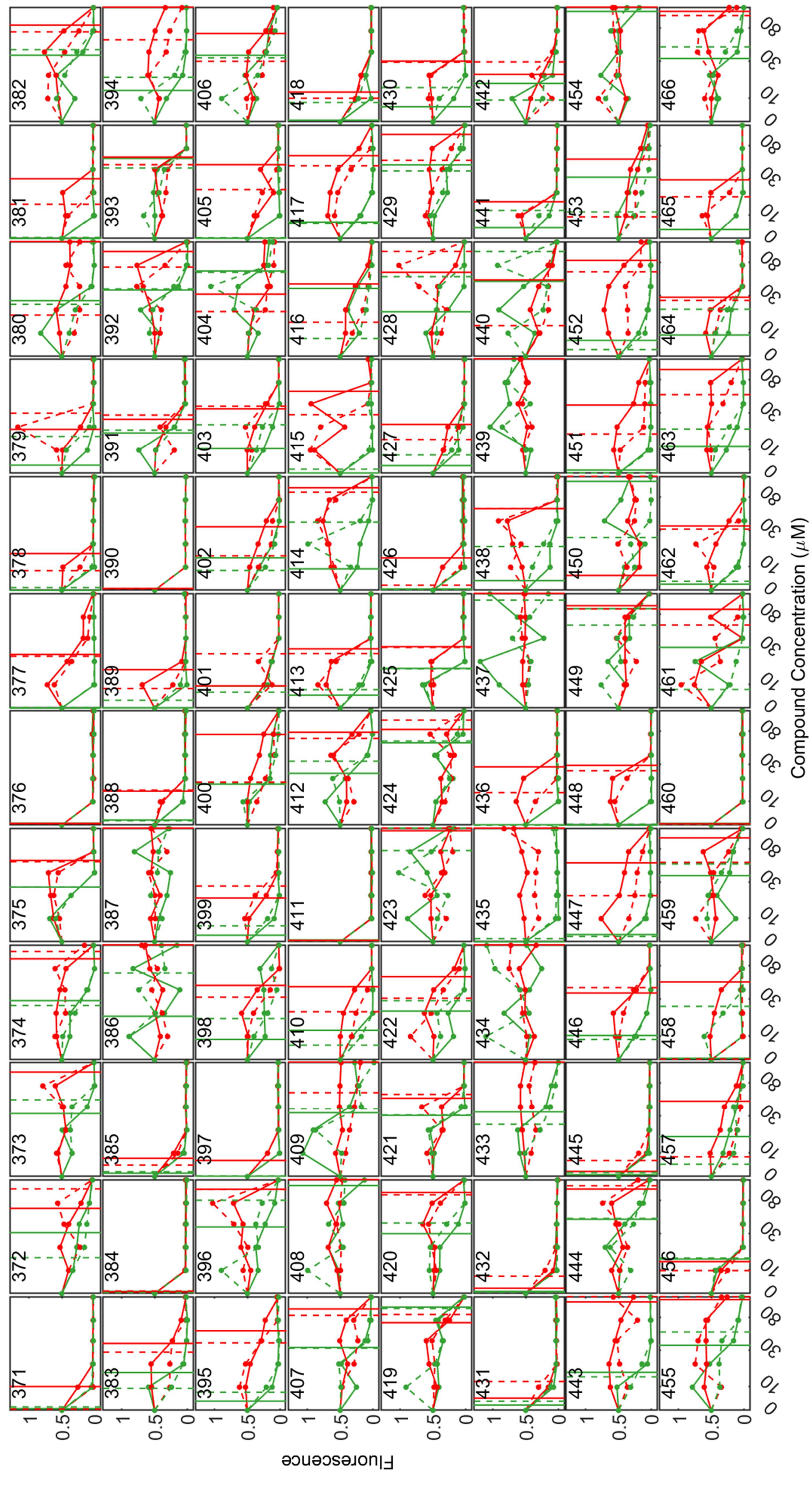

Supplementary Figure 5: Dose response curves and IC_{50}'s of all 1990 compounds screened in Dose-Response assay (7/22).

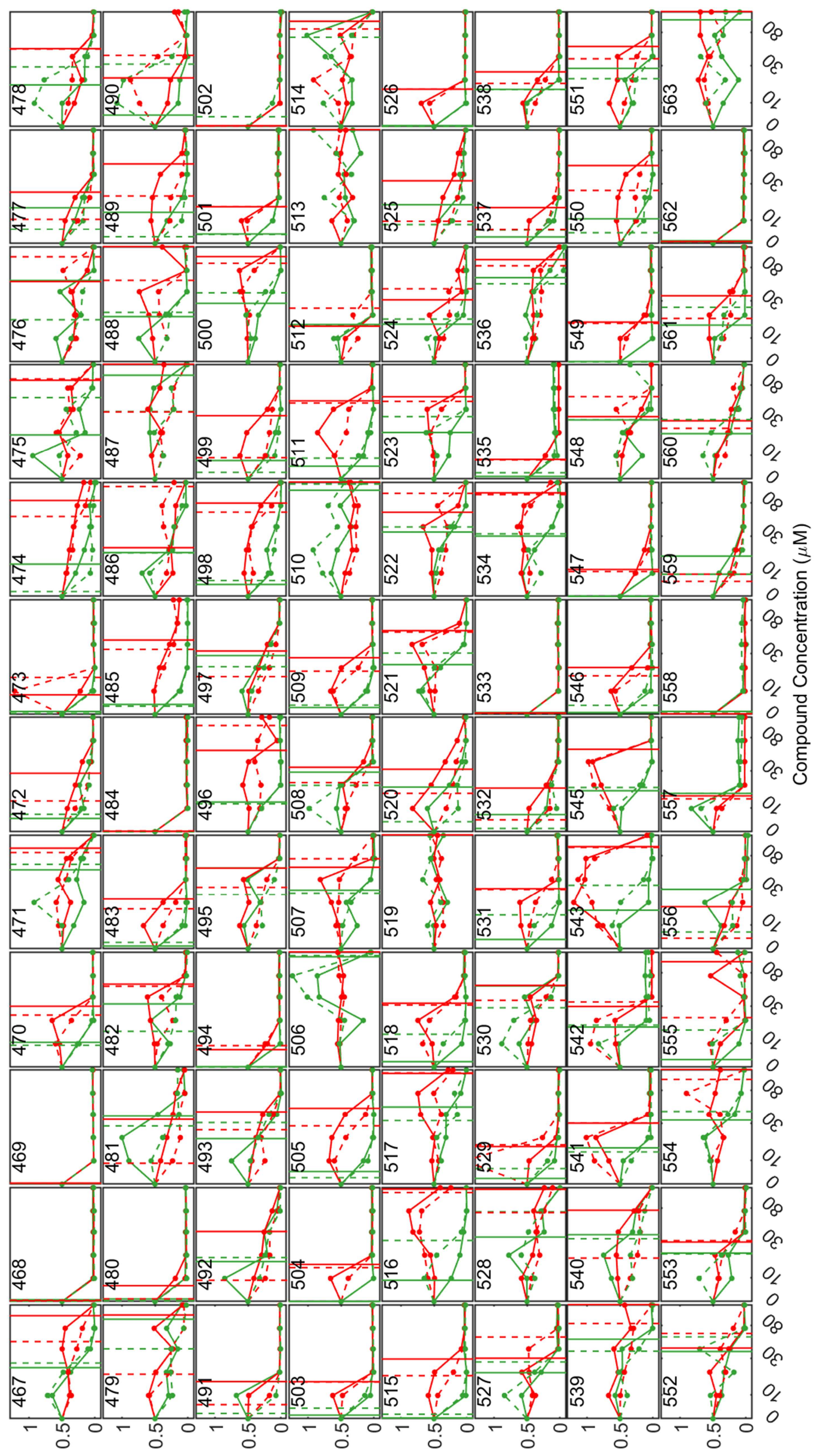

Supplementary Figure 5: Dose response curves and IC_{50}'s of all 1990 compounds screened in Dose-Response assay (8/22).

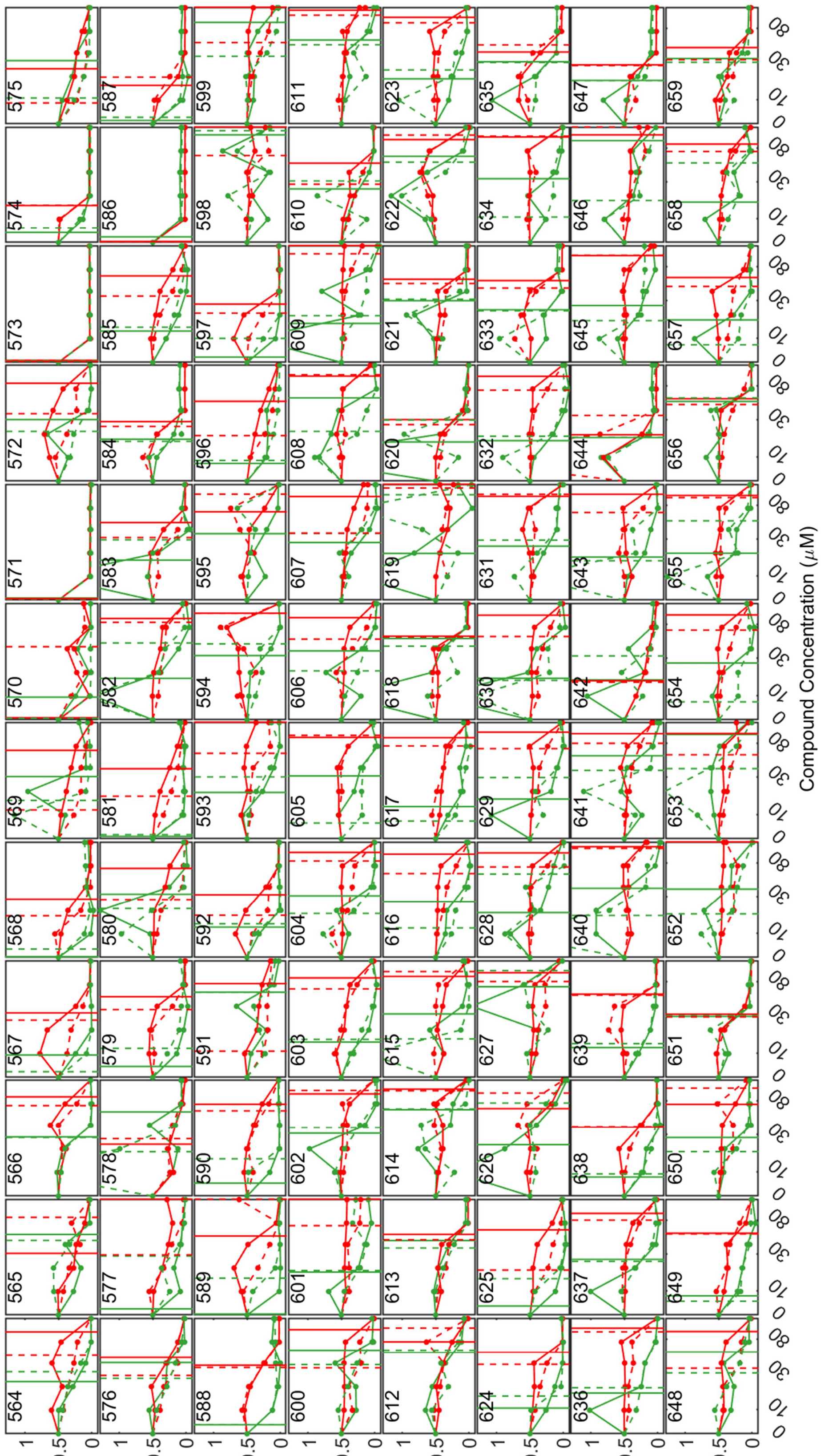

Supplementary Figure 5: Dose response curves and IC_{50}'s of all 1990 compounds screened in Dose-Response assay (9/22).

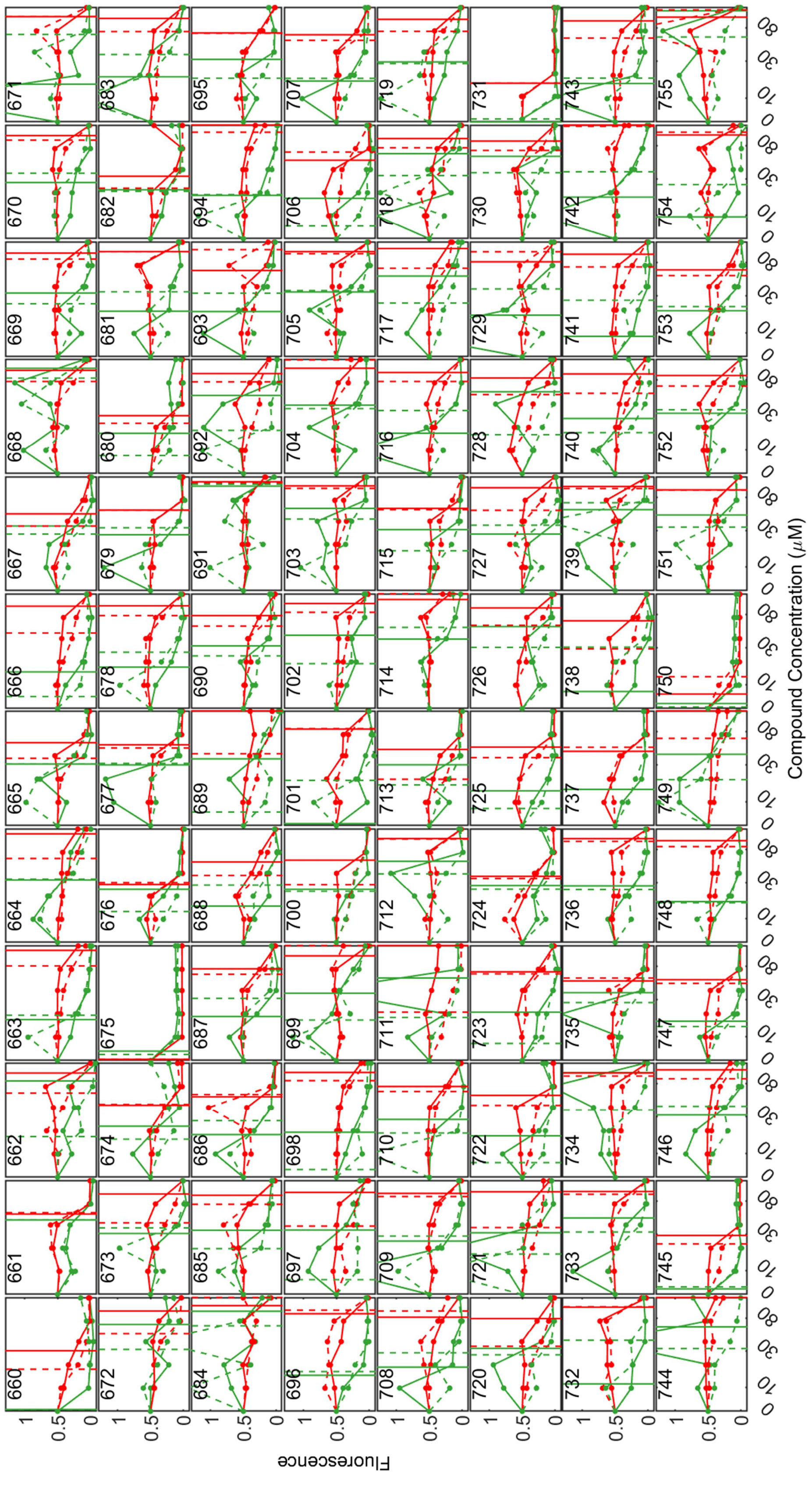

Supplementary Figure 5: Dose response curves and IC_{50}'s of all 1990 compounds screened in Dose-Response assay (10/22).

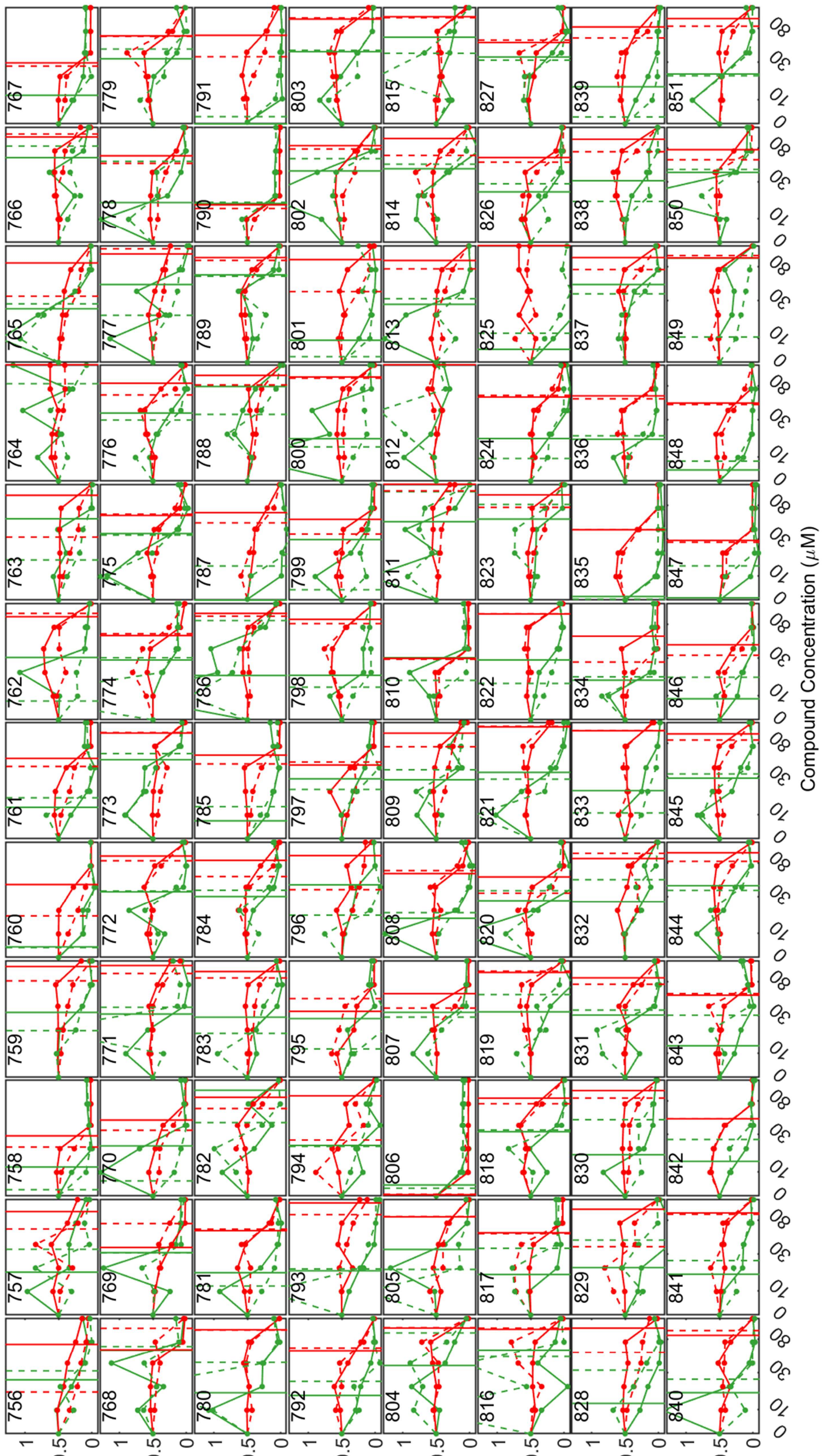

Supplementary Figure 5: Dose response curves and IC_{50}'s of all 1990 compounds screened in Dose-Response assay (11/22).

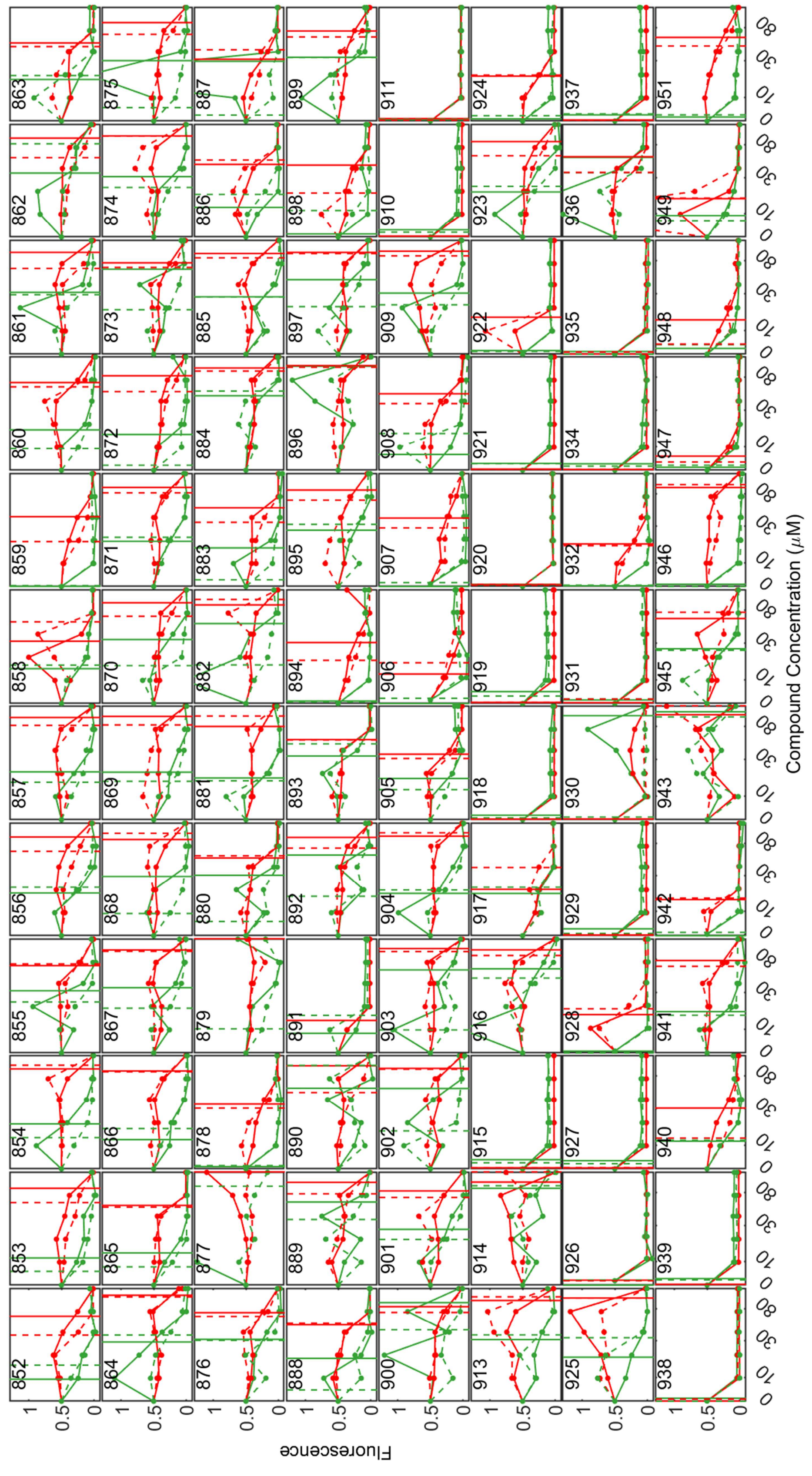

Supplementary Figure 5: Dose response curves and IC_{50}'s of all 1990 compounds screened in Dose-Response assay (12/22).

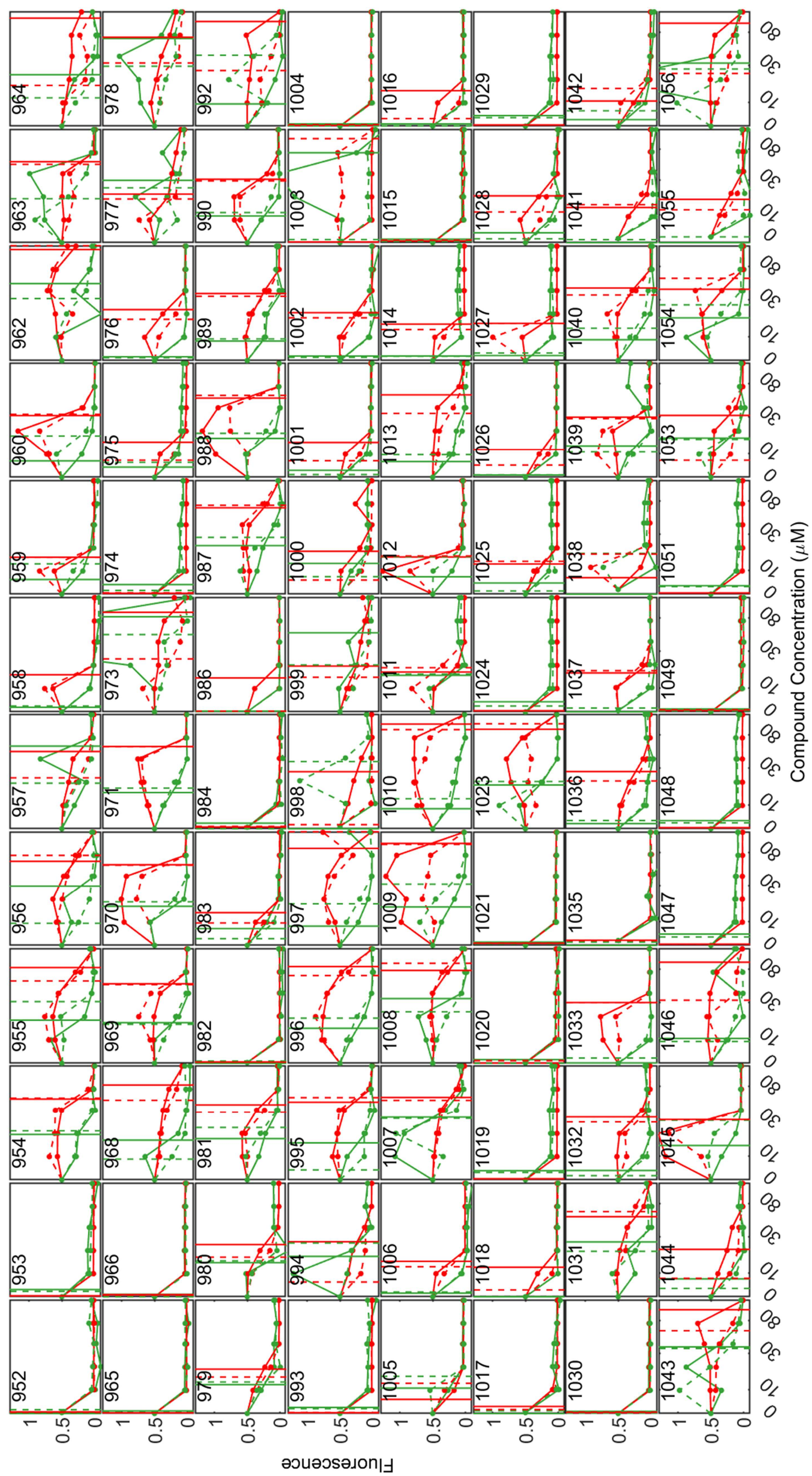

Supplementary Figure 5: Dose response curves and IC_{50}'s of all 1990 compounds screened in Dose-Response assay (13/22).

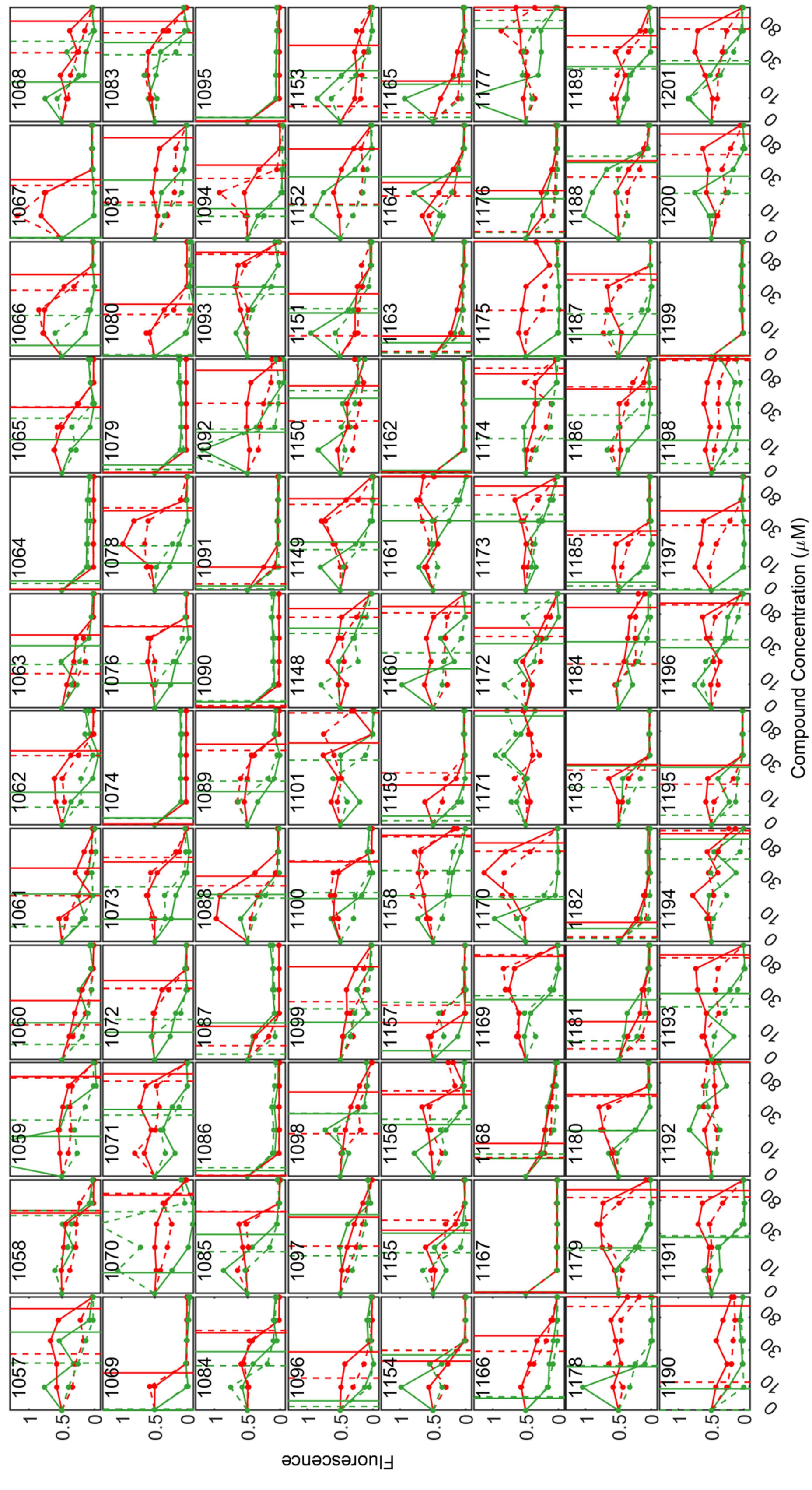

Supplementary Figure 5: Dose response curves and IC_{50}'s of all 1990 compounds screened in Dose-Response assay (14/22).

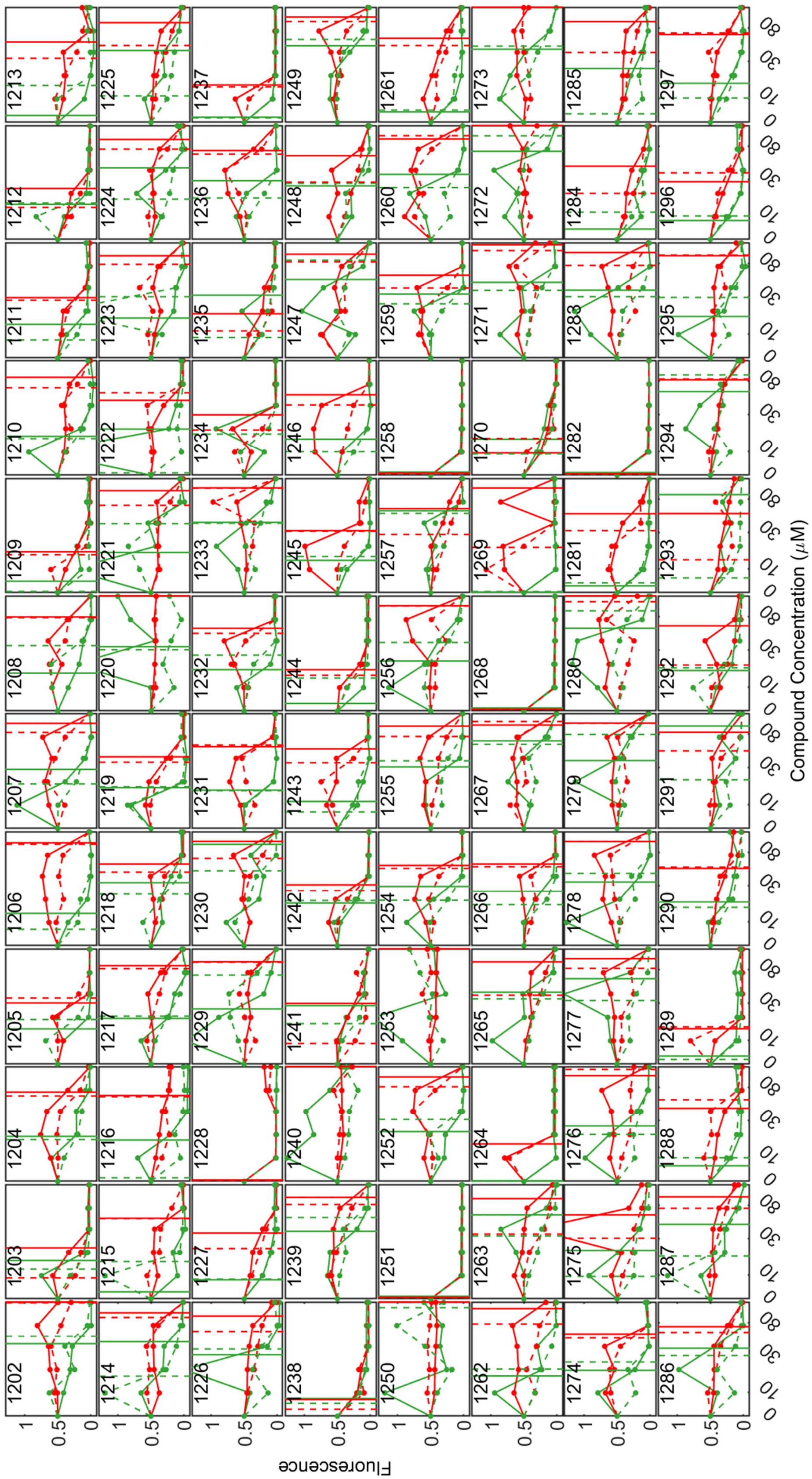

Supplementary Figure 5: Dose response curves and IC_{50}'s of all 1990 compounds screened in Dose-Response assay (15/22).

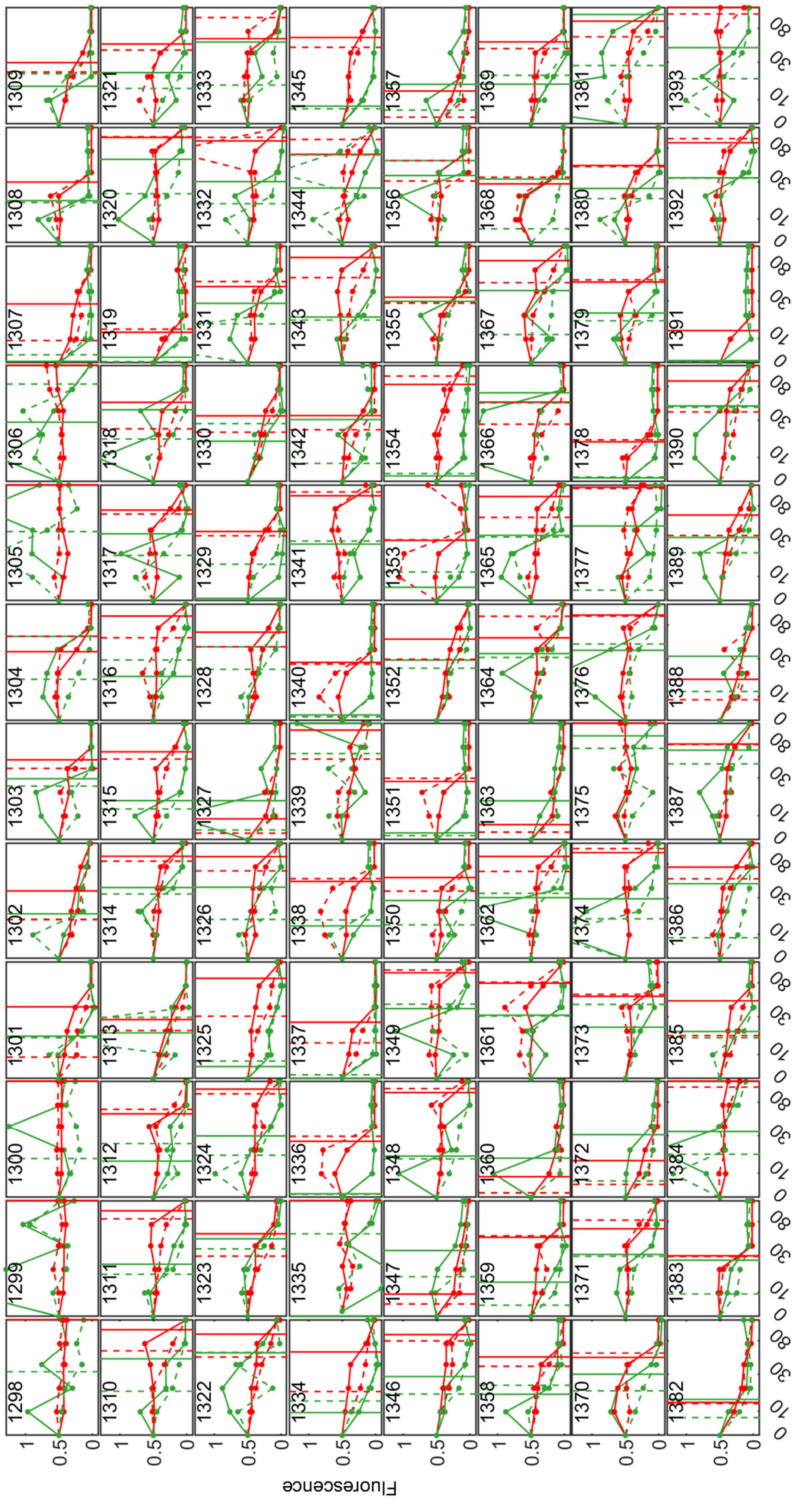

Supplementary Figure 5: Dose response curves and IC_{50}'s of all 1990 compounds screened in Dose-Response assay (16/22).

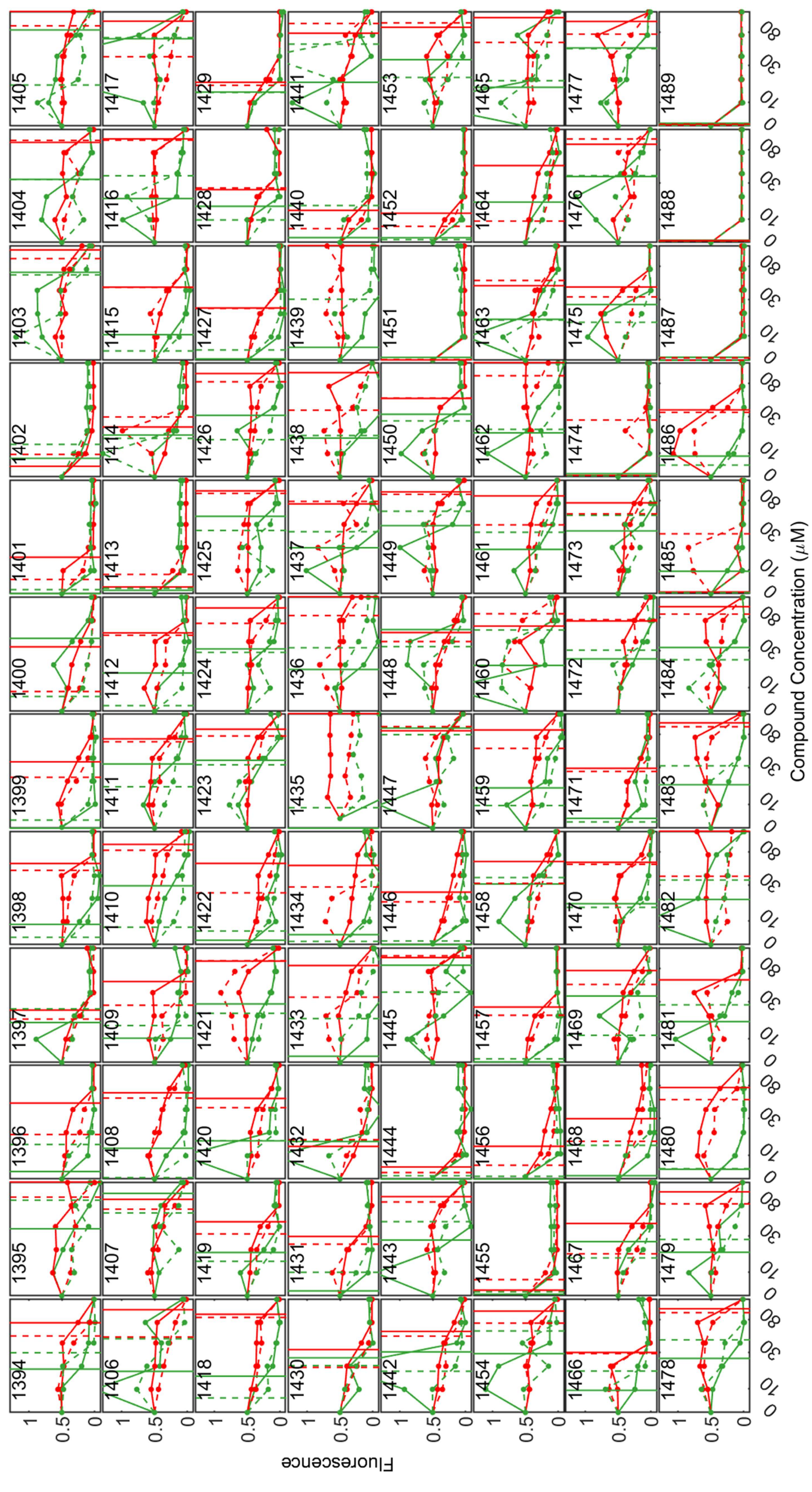

Supplementary Figure 5: Dose response curves and IC_{50}'s of all 1990 compounds screened in Dose-Response assay (17/22).

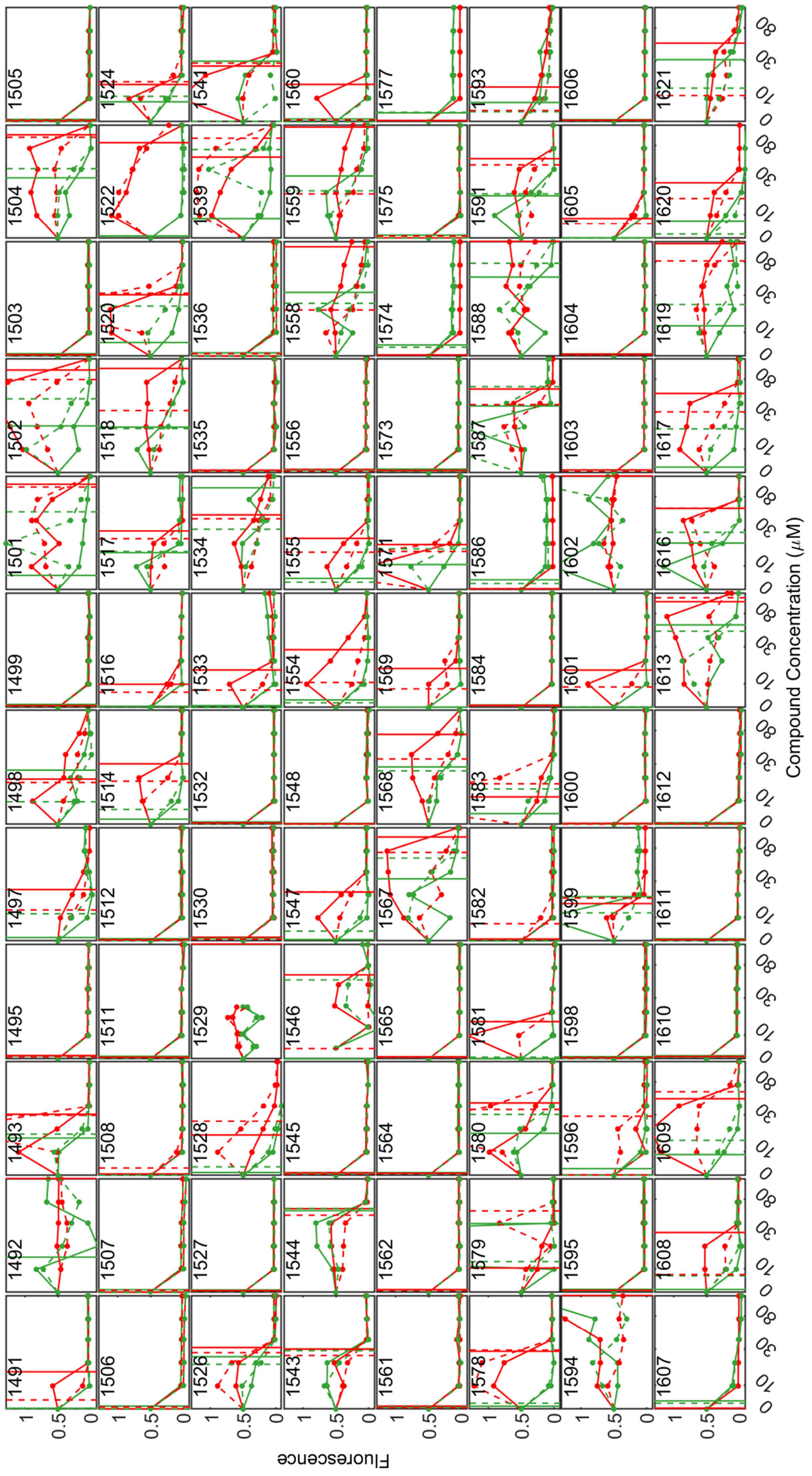

Supplementary Figure 5: Dose response curves and IC_{50}'s of all 1990 compounds screened in Dose-Response assay (18/22).

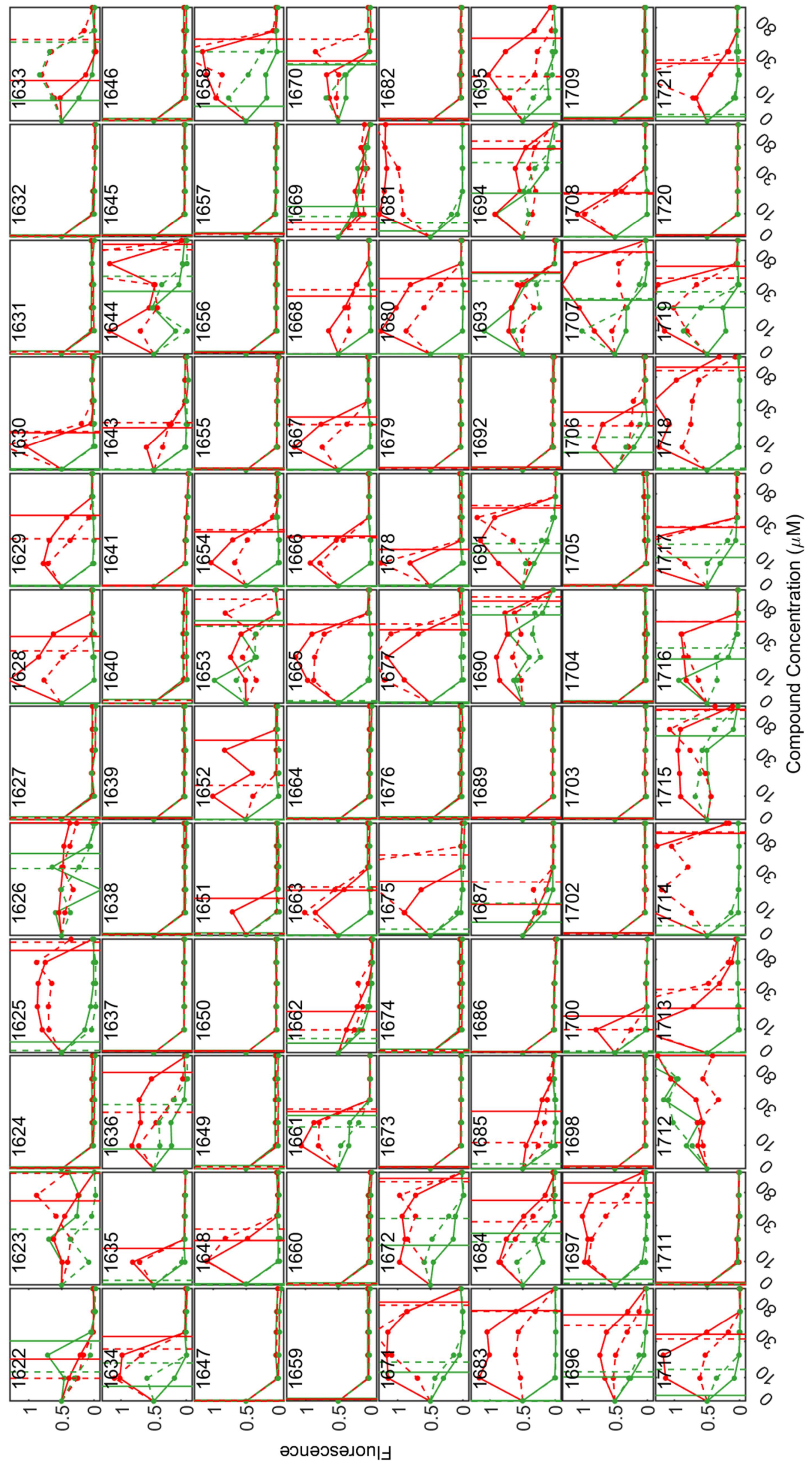

Supplementary Figure 5: Dose response curves and IC$_{50}$'s of all 1990 compounds screened in Dose-Response assay (19/22).

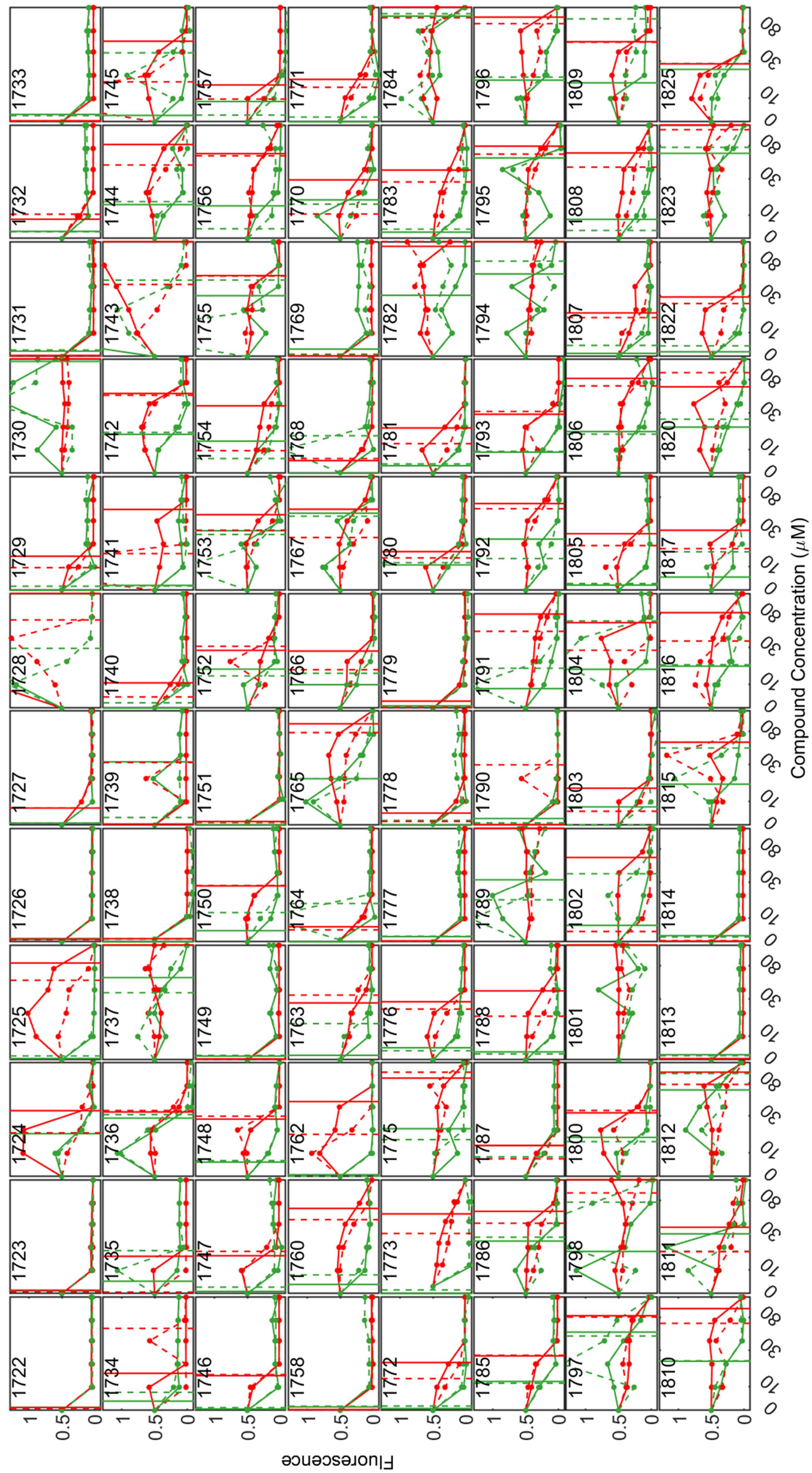

Supplementary Figure 5: Dose response curves and IC_{50}'s of all 1990 compounds screened in Dose-Response assay (20/22).

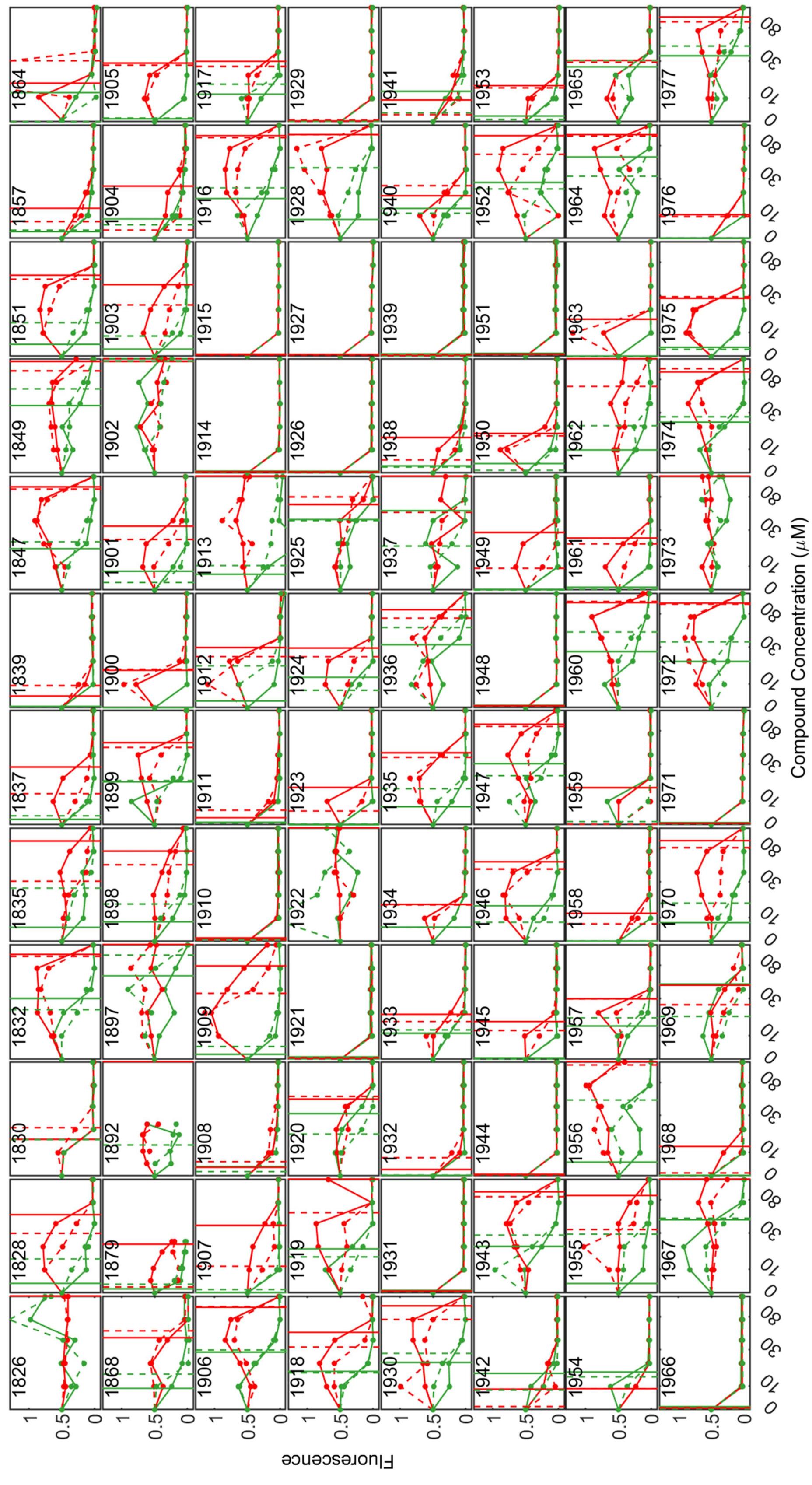

Supplementary Figure 5: Dose response curves and IC_{50}'s of all 1990 compounds screened in Dose-Response assay (21/22).

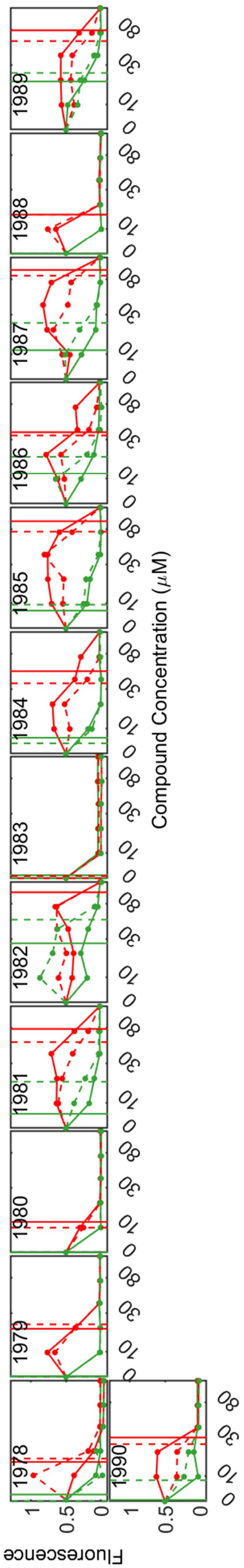

Supplementary Figure 5: Dose response curves and IC_{50}'s of all 1990 compounds screened in Dose-Response assay (22/22). Solid lines represent mecA+ (DsRed) and mecA- (GFP) strains. Dashed lines represent the dye-swap strains, mecA+ (GFP) and mecA- (DsRed). Vertical lines are the calculated IC50's of each strain (**see Supplementary Data 2** for calculated IC_{50}s, n = 2 dye-swapped replicates).

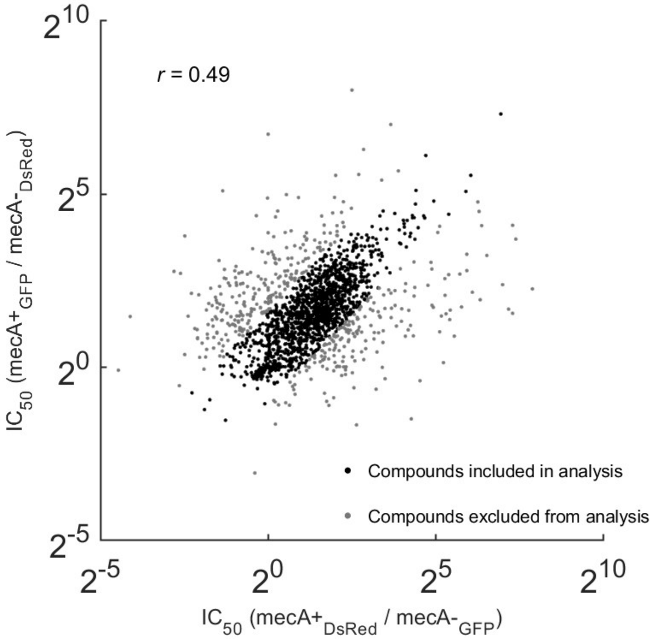

Supplementary Figure 6: Correlation between IC_{50}'s of dye-swap replicates in Dose-Response assay. Correlation between log2 ratio of mecA+ to mecA- strains IC_{50} dye-swap replicates of the 1,990 compounds tested in Dose-Response assay. Source data are provided as a Source Data file.

a

b

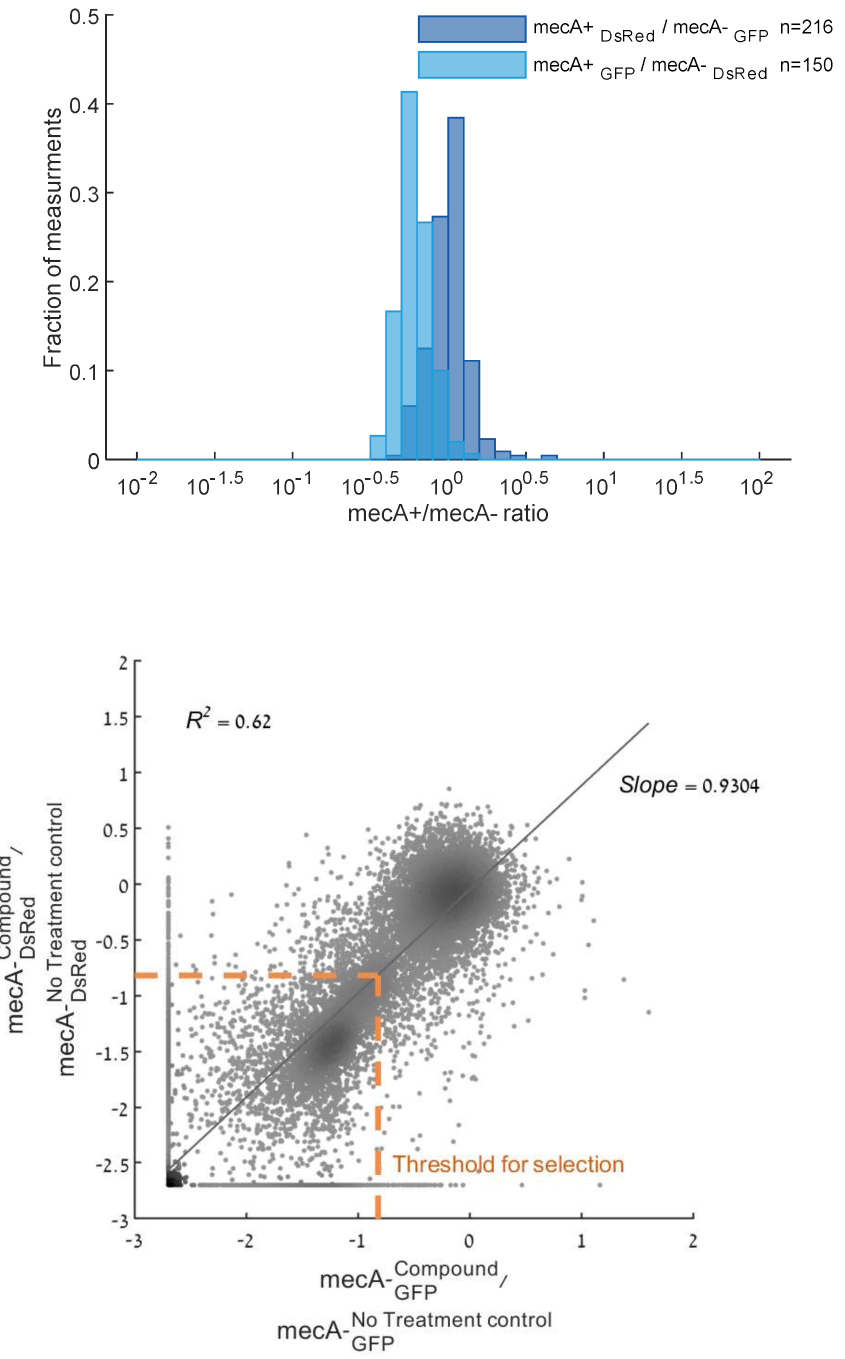

Supplementary Figure 7: **Assay analysis is not very sensitive to differential effects of the fluorescent markers. a,** Small cost of DsRed versus GFP fluorescent markers. Distribution of ratios of mecA+ to mecA- competing cells in the absence of any additional compounds (including cefoxitin). **b,** Fluorescent marker effects are minimized by defining selection relative to 'No Treatment' control of cells with the same fluorescent markers. Ratio between the selection coefficients of mecA+ (for each compound in each dye-swap assay, ratio between the mecA- fluorescent signal in the compound well to the mean signal of mecA- in the No Treatment control wells) is 0.93, signifying 7% mean deviation between the coefficients of the two fluorescent markers swaps, based on Dose-Response assay analysis (Methods). Source data are provided as a Source Data file.

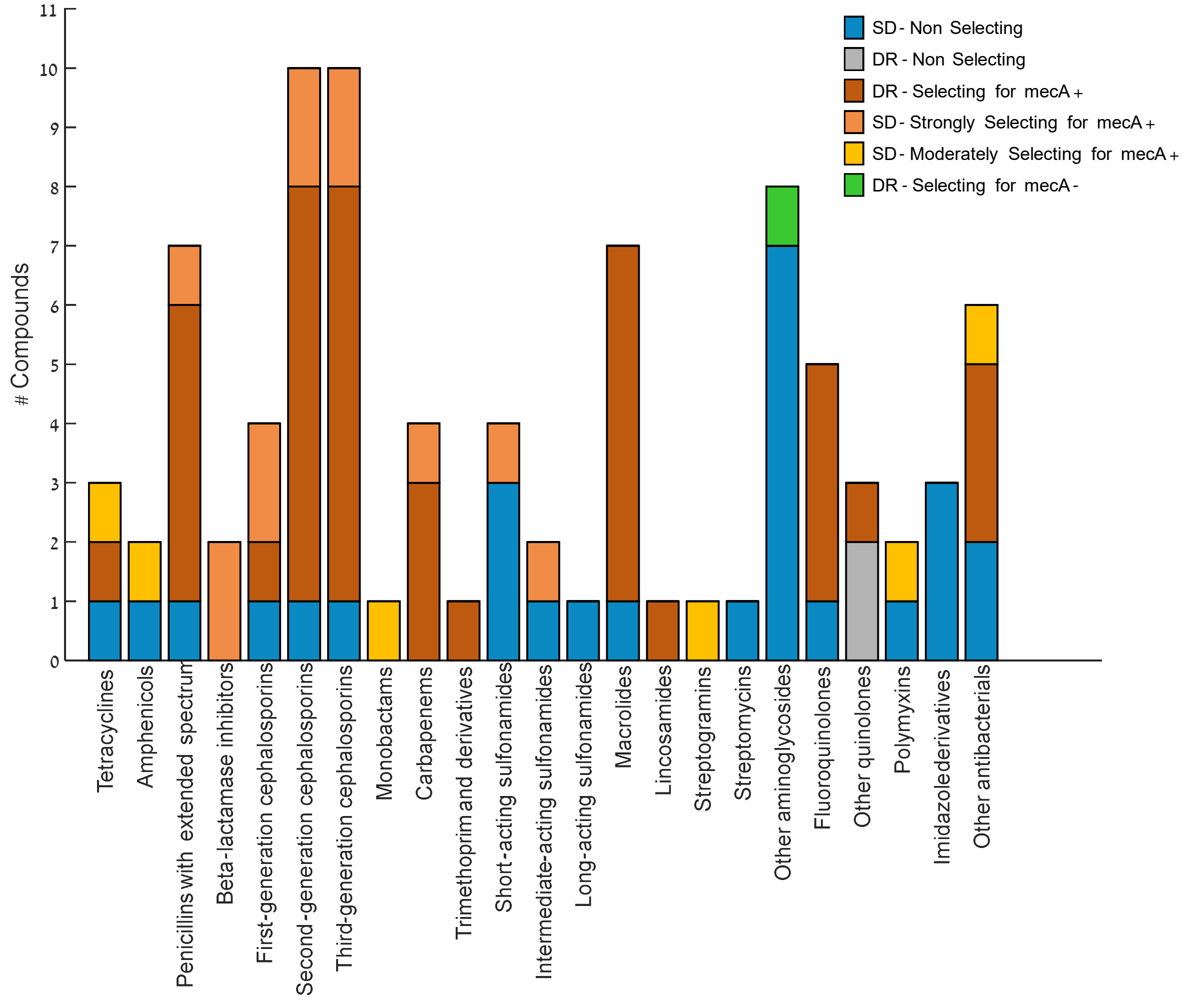

Supplementary Figure 8: Different antibiotic classes confer a selective advantage in favour of *mecA*. Almost all β-lactam antibiotics that were tested selected in favour of the mecA+ strain (few non-selective exceptions in the Single-Dose assay were likely a result of low dissolution in the screen, Methods). The selection in favour of the mecA+ strain was also conferred by many non-β-lactam antibiotics (n = 90 different antibiotics). SD- Single-Dose, DR- Dose-Response. Source data are provided as a Source Data file.

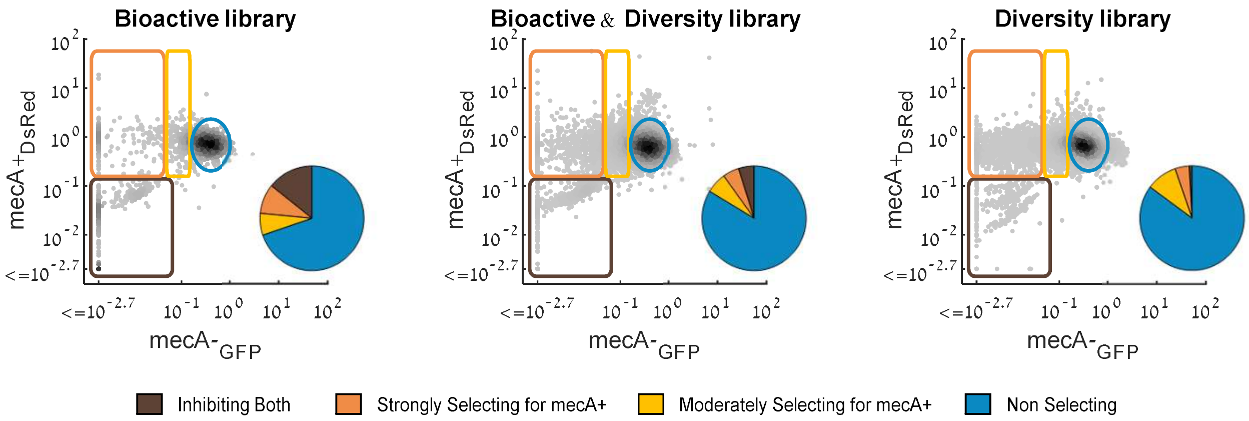

Supplementary Figure 9: Density plot of the normalized fluorescent signals for bioactive, diversity and bioactive & diversity library types. Test-compounds are separated into four groups by using thresholds determined by the median and standard deviation of the controls: non-selecting (blue), strongly and moderately selecting for mecA+ strain compounds (orange and yellow, respectively) and compounds inhibiting both strains (brown). The illustrated upper threshold for compounds moderately selecting for mecA+ is the mean of all the per-plate thresholds. Pie charts represent the frequency of each group within each of the libraries. Source data are provided as a Source Data file.

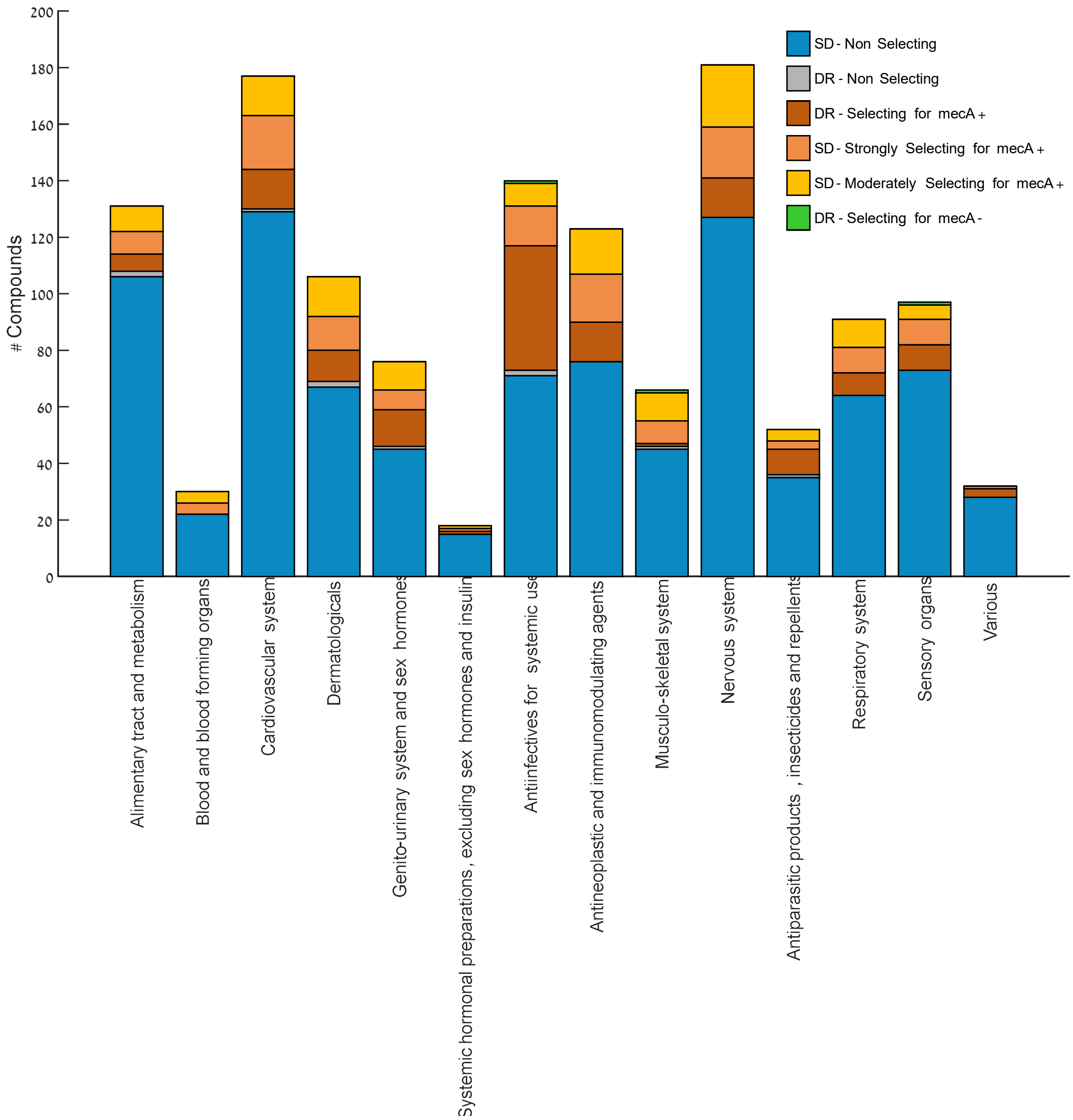

Supplementary Figure 10: Drugs belonging to all therapeutic drug groups confer a selective advantage in favour of *mecA*. All drug groups contain drugs that select in favour of *mecA* (n = 1,092 different drugs). Drugs belonging to more than one ATC drug group were counted once for each group. SD- Single-Dose, DR- Dose-Response. Source data are provided as a Source Data file.

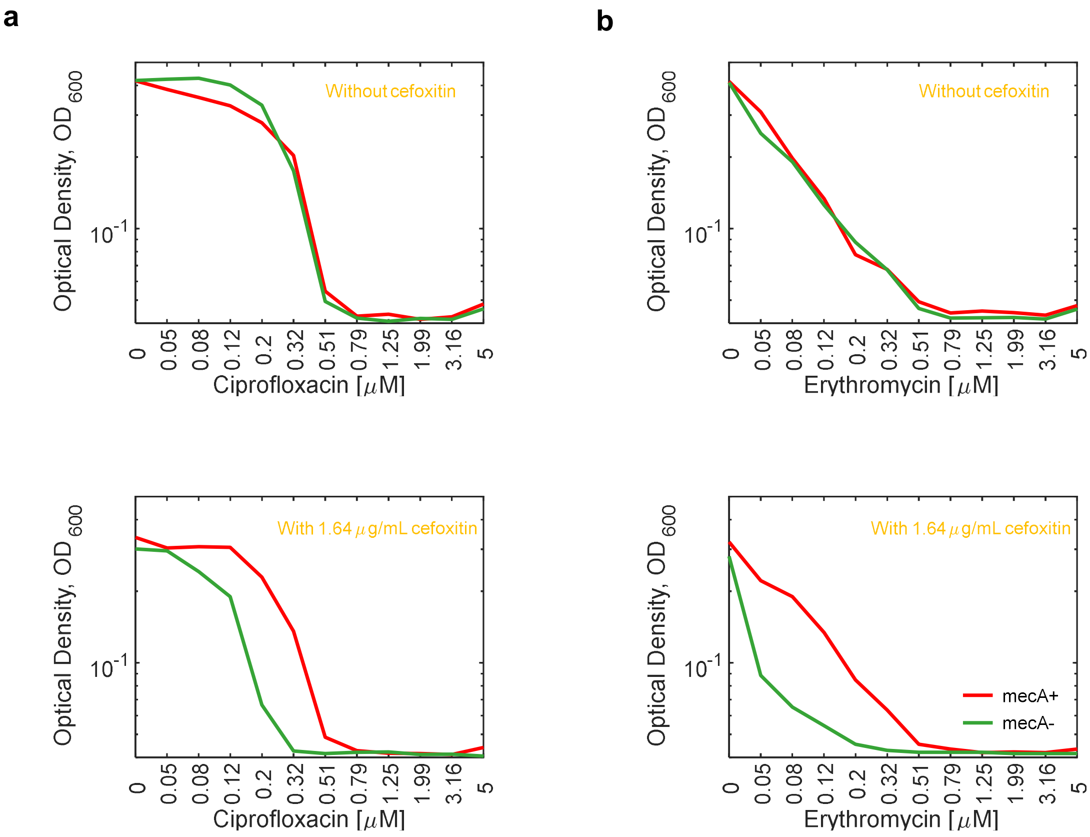

Supplementary Figure 11: Cefoxitin potentiates selection in favour of *mecA* by ciprofloxacin and erythromycin. OD_{600} as a function of ciprofloxacin or erythromycin concentration, with and without subinhibitory concentration of cefoxitin supplemented to the medium, after 10 hours of growth. Without cefoxitin, the MIC of each antibiotic is similar for both mecA+ and mecA- strains. When supplementing the medium with subinhibitory concentration of cefoxitin, the MIC of both ciprofloxacin and erythromycin is reduced for the mecA- strain only. Source data are provided as a Source Data file.

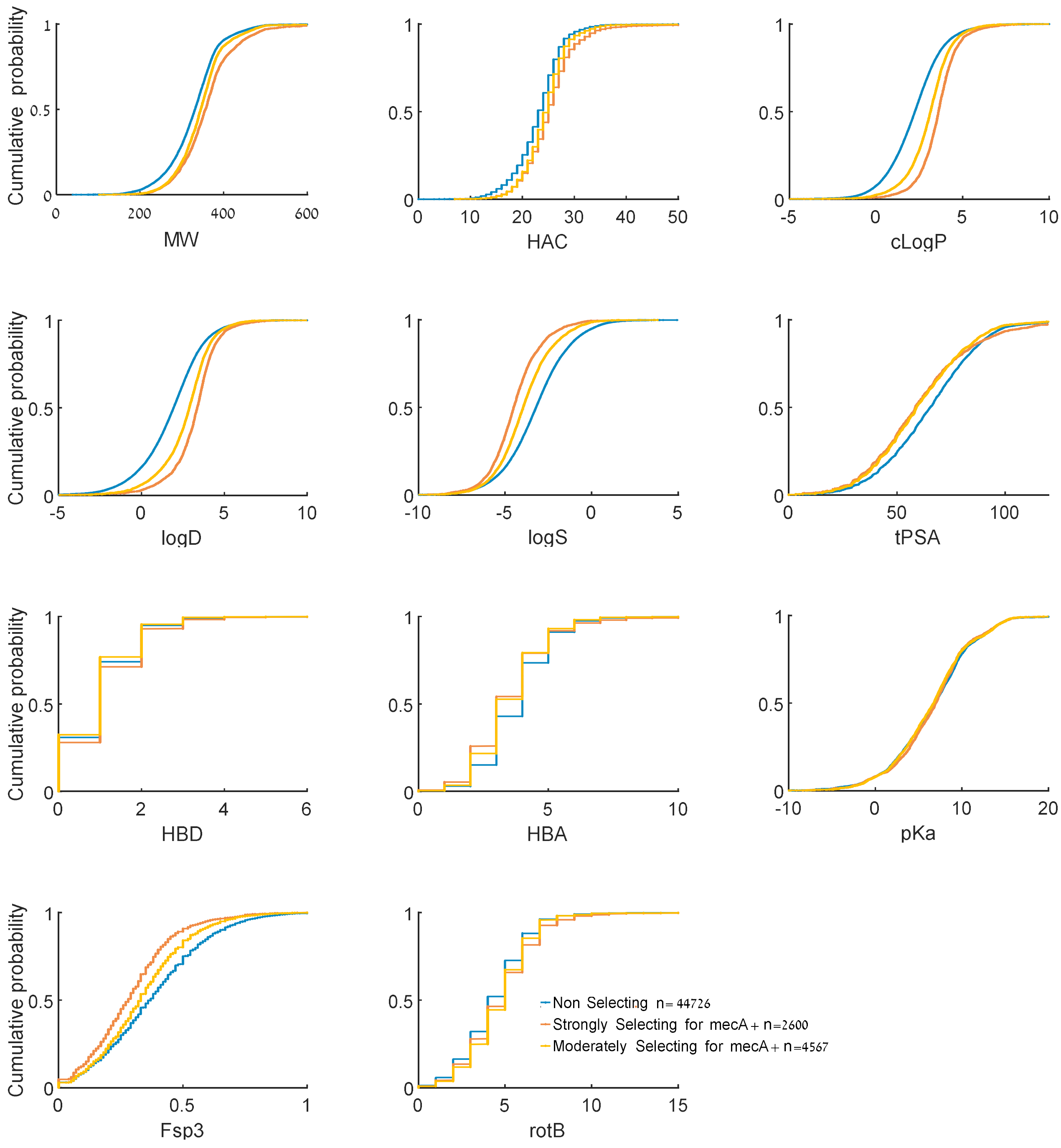

Supplementary Figure 12: Univariate correlations of chemical properties with strong selection in favour of *mecA* in Single-Dose assay. Cumulative distribution plots for all 11 properties tested. Source data are provided as a Source Data file.

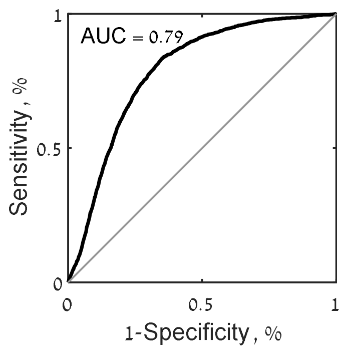

Supplementary Figure 13: Model performance for strong selection in favour of *mecA* by compounds' physicochemical properties. Performance of the logistic regression model for strong selection in favour of *mecA*. AUC indicates area under the receiver operating characteristic (ROC) curve. Source data are provided as a Source Data file.

Supplier	Library	Type	# Compounds
ChemBridge	DIVERSet"-CL	Diversity	51,227
Enamine	DLS	Diversity	20,307
MayBridge	HitFinder	Bioactive & Diversity	13,969
MicroSource	The Spectrum Collection	Bioactive & Diversity	2,392
Selleck Chemicals	L2000-Z107607, Bioactive Screening Libraries, Natural Products	Bioactive	2,190
SIGMA	LOPAC	Bioactive	1,277

Supplementary Table 1: Libraries screened.

Strain	Description	Methicillin resistance	Source
S. aureus MW2	MW2	Resistant	(67)
S. aureus Δ*mecA*	MW2Δ*mecA*	Sensitive	(67)
MW2 / pCM29-sGFP	Plasmid pCM29 (sarAP1_sGFP, cam^R) into MW2	Resistant	This study
MW2Δ*mecA* / pCM29-sGFP	Plasmid pCM29 (sarAP1_sGFP, cam^R) into MW2Δ*mecA*	Sensitive	This study
MW2 / pHC48-DsRed	Plasmid pHC48 (sarAP1_DsRed, cam^R) into MW2	Resistant	This study
MW2Δ*mecA* / pHC48-DsRed	Plasmid pHC48 (sarAP1_DsRed, cam^R) into MW2Δ*mecA*	Sensitive	This study

Supplementary Table 2: Strains used in the study.

Primer	Sequence (5’ → 3’)
mecA forward	CAGGAATGCAGAAAGACC
mecA reverse	GGAACGATGCCTATCTC′
gmk forward	CCATCTGGAGTAGGTAAAGG
gmk reverse	CTACGCCATCAACTTCAC

Supplementary Table 3: Primers used in the study.
