# Supplementary material for: Ubiquitous selection for mecA in community-associated MRSA across diverse chemical environments
Source: Nat Commun. 2020 Nov 27;11:6038. doi: 10.1038/s41467-020-19825-3 (PMC7695840; doi:10.1038/s41467-020-19825-3)
Supplement: Supplementary file 3 — Description of Additional Supplementary Files [file 41467_2020_19825_MOESM3_ESM.pdf]

Description of Additional Supplementary Files

File Name: Supplementary Data 1
Description: Raw GFP and DsRed signals for all compounds in the Single-Dose assay.

File Name: Supplementary Data 2
Description: Raw GFP and DsRed signals and calculated IC50's for all compounds in the Dose-Response assay.

File Name: Supplementary Data 3
Description: Non selecting and strongly selecting for mecA+ compounds' physicochemical properties.
