# Supplementary material for: Ubiquitous selection for mecA in community-associated MRSA across diverse chemical environments
Source: Nat Commun. 2020 Nov 27;11:6038. doi: 10.1038/s41467-020-19825-3 (PMC7695840; doi:10.1038/s41467-020-19825-3)
Supplement: Supplementary file 7 — Reporting Summary [file 41467_2020_19825_MOESM7_ESM.pdf]

nature research

Corresponding author(s): Roy Kishony

Last updated by author(s): Oct 2, 2020

Reporting Summary

Nature Research wishes to improve the reproducibility of the work that we publish. This form provides structure for consistency and transparency in reporting. For further information on Nature Research policies, see Authors & Referees and the Editorial Policy Checklist .

Statistics

For all statistical analyses, confirm that the following items are present in the figure legend, table legend, main text, or Methods section.

n/a	Confirmed	
☐	☒	The exact sample size (*n*) for each experimental group/condition, given as a discrete number and unit of measurement
☐	☒	A statement on whether measurements were taken from distinct samples or whether the same sample was measured repeatedly
☐	☒	The statistical test(s) used AND whether they are one- or two-sided<br>*Only common tests should be described solely by name; describe more complex techniques in the Methods section.*
☐	☒	A description of all covariates tested
☐	☒	A description of any assumptions or corrections, such as tests of normality and adjustment for multiple comparisons
☐	☒	A full description of the statistical parameters including central tendency (e.g. means) or other basic estimates (e.g. regression coefficient) AND variation (e.g. standard deviation) or associated estimates of uncertainty (e.g. confidence intervals)
☐	☒	For null hypothesis testing, the test statistic (e.g. *F*, *t*, *r*) with confidence intervals, effect sizes, degrees of freedom and *P* value noted<br>*Give P values as exact values whenever suitable.*
☒	☐	For Bayesian analysis, information on the choice of priors and Markov chain Monte Carlo settings
☒	☐	For hierarchical and complex designs, identification of the appropriate level for tests and full reporting of outcomes
☐	☒	Estimates of effect sizes (e.g. Cohen's *d*, Pearson's *r*), indicating how they were calculated

Our web collection on statistics for biologists contains articles on many of the points above.

Software and code

Policy information about availability of computer code

Data collection	The fluorescent signals were read by PHERAstar FS plate reader, software version 4.00 R4.
Data analysis	A custom MATLAB script (R2019a) was used to analyze the data, including analysis of the assay fluorescent signals, compound properties analysis, and all statistical tests. The script was also used to generate the figures and the tables. The custom script is publicly available on the lab website (https://kishony.technion.ac.il/resources/).

For manuscripts utilizing custom algorithms or software that are central to the research but not yet described in published literature, software must be made available to editors/reviewers. We strongly encourage code deposition in a community repository (e.g. GitHub). See the Nature Research guidelines for submitting code & software for further information.

Data

Policy information about availability of data

All manuscripts must include a data availability statement. This statement should provide the following information, where applicable:

- Accession codes, unique identifiers, or web links for publicly available datasets
- A list of figures that have associated raw data
- A description of any restrictions on data availability

All raw GFP and DsRed fluorescent signals of the mecA+ and mecA- strains in the Single-Dose assay are reported in Supplementary Table 2. All dose-response curves and calculated IC50's of the strains in the Dose-Response assay are reported in Supplementary Fig. 4 and Supplementary Table 1. All raw data is publicly available on the lab website (https://kishony.technion.ac.il/resources/).

Field-specific reporting

Please select the one below that is the best fit for your research. If you are not sure, read the appropriate sections before making your selection.

- [x] Life sciences
- [ ] Behavioural & social sciences
- [ ] Ecological, evolutionary & environmental sciences

For a reference copy of the document with all sections, see nature.com/documents/nr-reporting-summary-flat.pdf

Life sciences study design

All studies must disclose on these points even when the disclosure is negative.

Sample size	We screened 89,302 compounds in single high dose of 150 uM. Compounds that inhibited both strains (1,990) were screened in a concentration gradient in replicate with the fluorescent markers swapped.
Data exclusions	Median ratio of mecA+ (DsRed) to mecA- (GFP) in 'No Treatment' control wells in each platen was used to decide on inclusion or exclusion of test plates from analysis. Test plates with high ratio between the cells were excluded from analysis, as the high ratio may indicate that a too high cefoxitin concentration was used in the sub-screen batch, selecting for mecA+ strain even in the absence of other compounds Systematically high ratio of normalized DsRed/GFP fluorescent signal (DsRed/GFP>10) was used to threshold out rows having dispensing errors. These data were excluded from all further analysis.
Replication	To assess and verify reproducibility, we have conducted a Pilot assay, prior to the large-scale assays, screening 1193 compounds in replicates. All attempts at replication with similar cefoxitin concentration were successful.
Randomization	Randomization was not relevant to this study, all samples were analyzed equally.
Blinding	All fluorescent measurements of the cells in the presence of different compounds were analyzed in the same way, while blind to the compound identity of in each well.

Reporting for specific materials, systems and methods

We require information from authors about some types of materials, experimental systems and methods used in many studies. Here, indicate whether each material, system or method listed is relevant to your study. If you are not sure if a list item applies to your research, read the appropriate section before selecting a response.

Materials & experimental systems

n/a	Involved in the study	
x		Antibodies
x		Eukaryotic cell lines
x		Palaeontology
x		Animals and other organisms
x		Human research participants
x		Clinical data

Methods

n/a	Involved in the study	
x		ChIP-seq
x		Flow cytometry
x		MRI-based neuroimaging
